# Discovery of ianthelliformisamines D–G from the sponge *Suberea ianthelliformis* and the total synthesis of ianthelliformisamine D

**DOI:** 10.3762/bjoc.20.266

**Published:** 2024-12-09

**Authors:** Sasha Hayes, Yaoying Lu, Bernd H A Rehm, Rohan A Davis

**Affiliations:** 1 Institute for Biomedicine and Glycomics, Griffith University, Don Young Road, Brisbane, 4111, Australia; 2 NatureBank, Griffith University, Don Young Road, Brisbane, 4111, Australia; 3 Centre for Cell Factories and Biopolymers, Griffith University, Don Young Road, Brisbane, 4111, Australia

**Keywords:** ianthelliformisamine, marine sponge, natural products, *Pseudomonas*, *Suberea*, total synthesis

## Abstract

The marine sponge *Suberea ianthelliformis* was investigated for new chemistry after the recent discovery that polyamines ianthelliformisamines A–C (**1**–**3**) – originally sourced from this Australian sponge – act as *Pseudomonas aeruginosa* biofilm inhibitors and antibiotic enhancers. Large-scale extraction and isolation studies resulted in the discovery of four new and minor natural products, ianthelliformisamines D–G (**4**–**7**) and the known steroid, aplysterol (**8**). Compounds **4**–**7** were fully characterised following 1D/2D NMR, MS and UV data analyses. All compounds were assessed for their inhibition on planktonic growth of *P. aeruginosa* PAO1 in addition to their ability to inhibit the formation of biofilms. None of the tested natural products inhibited planktonic growth or biofilm formation of PAO1 when screened at 50 µM. Ianthelliformisamine D (**4**) contains a rare *N*-(3-aminopropyl)-2-pyrrolidone moiety only found in <30 natural products. Owing to the novelty of compound **4**, we undertook the first total synthesis of this natural product, which was achieved in three steps.

## Introduction

The marine environment covers over two thirds of the earth’s surface and it encompasses a wide range of complex ecosystems that are highly variable in their physical attributes including pressure, salinity, temperature, and light availability. Both flora and fauna have evolved over billions of years to survive within this unique environment [1–2], which has led to the production and diversification of unique and novel metabolites with specialised biological functions [3–4]. The richest diversity of marine metabolites are derived from invertebrates; most of which are sessile and lack the ability to physically defend themselves from both predators and competitors and thus rely on chemical mechanisms for their defense [1]. Metabolites derived from marine sponges contribute more than half of all compounds identified from marine invertebrates [1,5], therefore it is no surprise that sponges are highly sought after for novel bioactive metabolites and have been a major focus of marine natural product drug discovery for over 70 years.

The identification of ianthelliformisamines A–C from the Australian marine sponge *Suberea ianthelliformis*, which displayed activity against both *Pseudomonas aeruginosa* and *Staphylococcus aureus,* has contributed to a surge in the interest of polyamines as new antibacterial leads [6]. To date the total synthesis of ianthelliformisamines A–C (**1**–**3**) has been described [7] and numerous synthetically related analogues have been published with their antibiotic assessment against numerous bacterial species reported [8–11]. Our recent publication showed that of the naturally occurring metabolites, compound **3** inhibits the formation of *P. aeruginosa* PAO1 biofilms (MIC 53.1 µg/mL), whilst **1** and **2** enhanced the antibiotic effect of ciprofloxacin when used in combination [12]. In efforts to further examine the chemistry of *S. ianthelliformis* and potentially discover new antibiotic leads, we undertook a scaled-up chemical investigation on the original specimen from which ianthelliformisamines A–C were isolated.

Herein, we describe the large-scale extraction, isolation, and structure elucidation of four new metabolites, ianthelliformisamines D–G (**4**–**7**). Additionally, we report the isolation of the known natural products, aplysterol (**8**) and ianthelliformisamines A–C (**1**–**3**). Owing to the novelty of ianthelliformisamine D (**4**) we undertook the first total synthesis of this natural product, which was successfully achieved in only three steps and respectable yield. All newly identified metabolites were subsequently assessed for their ability to inhibit the growth of planktonic *P. aeruginosa* PAO1 and formation of biofilms.

## Results and Discussion

**Figure 1:**
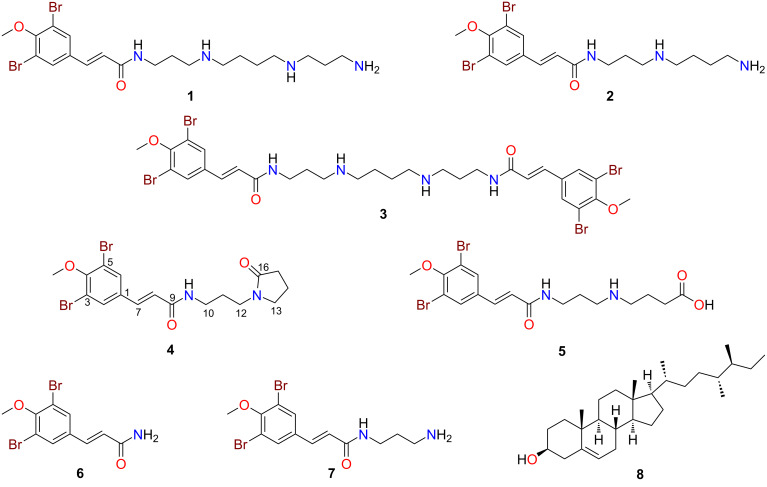
Chemical structures of ianthelliformisamines A–G (**1**–**7**) and aplysterol (**8**).

For a more comprehensive chemical investigation into the chemistry of *Suberea ianthelliformis*, a new aliquot of the freeze-dried and ground sample was extracted exhaustively with *n-*hexane, CH_2_Cl_2_, and MeOH. The CH_2_Cl_2_ and MeOH extracts were combined then subjected to phenyl-bonded reversed-phase HPLC (RP-HPLC), which led to the purification of the known metabolites, ianthelliformisamines A–C (**1**–**3**) [7] and aplysterol (**8**) [13], and four new natural products, ianthelliformisamines D–G (**4**–**7**) (Figure 1). This extraction and isolation process was repeated twice to obtain sufficient quantities of the minor and previously undescribed natural products for full characterisation studies and biological assessment. The spectroscopic and spectrometric data of all known compounds isolated during our studies matched well with the literature values [7,14].

**Figure 2:**
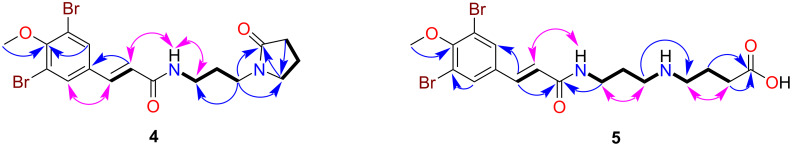
Key COSY (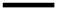), HMBC (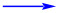) and ROESY (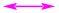) correlations for ianthelliformisamines D (**4**) and E (**5**).

Ianthelliformisamine D (**4**) was isolated as a stable brown gum. The LRESIMS of **4** indicated the presence of two bromine atoms due to a 1:2:1 ion cluster at *m/z* 459/461/463 [M + H]^+^_,_ whilst the HRESIMS data allowed a molecular formula of C_17_H_20_Br_2_N_2_O_3_ to be assigned. The ^1^H NMR (Table 1) and edited HSQC spectra of **4** in DMSO-*d*_6_ indicated the presence of one methoxy group (δ_H_ 3.81), six methylene signals (δ_H_ 3.33, 3.20, 3.14, 2.21, 1.91, 1.63) and one exchangeable proton triplet (δ_H_ 8.04) that was indicative of a secondary amide [7]. Additionally, signals for one isolated *trans* olefin (δ_H_ 7.33, 6.66) and one aromatic signal (δ_H_ 7.89) that integrated for two protons indicating the presence of a symmetrical aromatic moiety, were observed. The ^13^C NMR (Table 1) spectrum of **4** showed two carbonyls (δ_C_ 164.4, 173.9), with the carbonyl at δ_C_ 164.4 readily assigned to an acrylamide group, which is present in all previously published ianthelliformisamine molecules [7]. Methylenes resonating at δ_H_ 3.14, 1.64 and 3.20 were assigned to a three-carbon alkyl spin system that was flanked by nitrogen atoms [NH–(CH_2_)_3_–N] based on COSY correlations from the amide proton at δ_H_ 8.04. A terminal pyrrolidone was assigned based on COSY data for the three remaining methylene protons (δ_H_ 3.33, 1.91, 2.21), and a three-bond HMBC correlation from δ_H_ 3.33, and a two-bond correlation from δ_H_ 2.20 to the carbonyl resonating at δ_C_ 173.9 [15]. The observation of a ^3^*J*_CH_ correlation from δ_H_ 3.20 to the nitrogen-bearing carbon at δ_C_ 46.4 and to δ_C_ 173.9 supported the assignment of the pyrrolidone moiety. A 1,3,4,5-tetrasubstituted aromatic ring was assigned based on ^3^*J*_CH_ correlations from the isolated aromatic signal (δ_H_ 7.89) to its symmetrically placed carbon (δ_C_ 131.6) and to an oxygen-bearing carbon (δ_C_ 153.9) along with ^2^*J*_CH_ correlations to a brominated (δ_C_ 118.0) and sp^2^ phenyl carbons (*δ*_C_ 134.5) (see Figure 2). Observation of three-bond correlations in the HMBC spectrum between the olefin protons and aromatic ring carbons (δ_H_ 7.33 to δ_C_ 131.6 and δ_H_ 6.66 to δ_C_ 134.5) linked the olefin moiety to the symmetrical 1,3,4,5-tetrasubstituted phenyl system. ROESY and HMBC correlations from the amide proton linked the propyl-2-pyrrolidine moiety to the brominated acrylamide fragment and thus the full chemical structure of **4** was elucidated and assigned to ianthelliformisamine D. A refined literature search using Scifinder Scholar [16] revealed that compound **4** contains a novel scaffold due to the *N*-(3-aminopropyl)-2-pyrrolidone moiety, which is rarely found in natural products with <30 metabolites reported to date.

**Table 1:** NMR data of ianthelliformisamines D (**4**) and E (**5**) in DMSO-*d*_6_.^a^

	Ianthelliformisamine D (**4**)			Ianthelliformisamine E (**5**)^b^	
			
position	δ_C_, type	δ_H_, mult. (*J* in Hz)		δ_C_, type	δ_H_, mult. (*J* in Hz)

1	134.5, C			134.3, C	
2	131.6, CH	7.89, s		131.6, CH	7.89, s
3	118.0, C			118.0, C	
4	153.9, C			153.9, C	
4-OCH_3_	60.6, CH_3_	3.81, s		60.6, CH_3_	3.82, s
5	118.0, C			118.0, C	
6	131.6, CH	7.89, s		131.6, CH	7.89, s
7	135.2, CH	7.33, d (15.8)		135.5, CH	7.36, d (15.8)
8	124.5, CH	6.66, d (15.8)		124.1, CH	6.65, d (15.8)
9	164.4, C			164.9, C	
9-NH		8.04, t (5.7)			8.23, t (5.7)
10	36.5, CH_2_	3.14 dt (5.7, 6.5)		35.9, CH_2_	3.25 dt (5.7, 6.5)
11	27.0, CH_2_	1.63, m		26.1, CH_2_	1.77, m
12	39.7, CH_2_	3.20, t (7.2)		44.8, CH_2_	2.92, m
12-NH					8.35, brs
13	46.4, CH_2_	3.33, m		46.3, CH_2_	2.92, m
14	17.5, CH_2_	1.91, m		21.1, CH_2_	1.79, m
15	30.5, CH_2_	2.21, t (8.0)		30.4, CH_2_	2.36, t (7.3)
16	173.9, C			173.6, C	
16-OH					^c^

^a^Spectra recorded at 25 °C (800 MHz for ^1^H NMR and 200 MHz for ^13^C NMR); ^b^isolated as a TFA salt; ^c^not observed.

The TFA salt of ianthelliformisamine E (**5**) was obtained as a stable brown gum. The LRESIMS of **5** indicated the presence of two bromine atoms, displaying a 1:2:1 ion cluster at *m*/*z* 477/479/481 [M + H]^+^. Similarly to compound **4**, the ^1^H NMR (Table 1) and edited HSQC spectra of **5** displayed six methylene signals (δ_H_ 3.25, 2.92, 2.36, 1.79, 1.77). Additionally, a broad exchangeable proton (δ_H_ 8.35) was observed that was indicative of a protonated dialkylated amino group [7]. The ^13^C NMR (Table 1) data of **5** displayed six aliphatic carbons (δ_C_ 35.9, 26.1, 44.8, 46.3, 21.1, 30.4) and two carbonyl signals (δ_C_ 164.9, 173.6). Similarly to the other ianthelliformisamines, the aromatic (δ_C_ 131.6) and olefin (δ_C_ 124.5, 135.2) carbons were observed [7]. COSY correlations associated with the methylene signals of **5** enabled the assignment of an NH–(CH_2_)_3_–NH–(CH_2_)_3_ spin system, which was confirmed by HMBC and ROESY correlations. The carboxyl group at δ_C_ 173.6 was positioned at the end of the alkyl chain based on HMBC correlations from the methylene protons (δ_H_ 2.36, 1.79) to this carbon signal. Although a downfield exchangeable CO_2_H proton was not observed in the ^1^H NMR spectrum of **5**, a carboxylic acid moiety was assigned based on the ^13^C NMR shift value (δ_C_ 173.6) [17], and analysis of the HRESIMS ion at *m*/*z* 477.0022 [M + H]^+^, which confirmed the molecular formula to be C_17_H_22_Br_2_N_2_O_4_.

Ianthelliformisamine F (**6**) was isolated as a stable brown gum. The LRESIMS of **6** indicated the presence of two bromine atoms due to a 1:2:1 ion cluster at *m*/*z* 334/336/338 [M + H]^+^, whilst the HRESIMS data enabled a molecular formula of C_10_H_9_Br_2_NO_2_ to be assigned. Comparison of the 1D NMR data of **6** to metabolites **4** and **5** and the previously characterised ianthelliformisamines A–C readily allowed the assignment of dibromo-4-methoxyphenyl and propenamide moieties. However, the ^1^H NMR spectrum of **6** displayed two broad exchangeable signals (δ_H_ 7.19, 7.46) indicating the presence of a primary amide [18], thus enabling the full chemical structure of ianthelliformisamine F to be determined.

**Figure 3:**
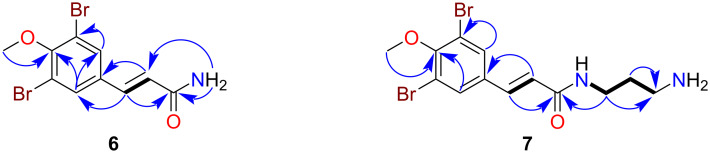
Key COSY (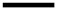) and HMBC (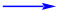) correlations for ianthelliformisamines F (**6**) and G (**7**).

The TFA salt of ianthelliformisamine G (**7**) was also purified as a stable brown gum. In a similar manner to compound **6**, the LRESIMS indicated the presence of two bromine atoms; these data also indicated that **7** had an additional 57 amu (atomic mass units) compared to **6**. Comparison of the ^1^H NMR (Table 2) and edited HSQC spectra with **7** showed that **6** contained three additional methylene signals (δ_H_ 3.25, 2.81, 1.73). Two exchangeable protons were observed in **7**, including a broad singlet (δ_H_ 7.73) that integrated for three protons and a triplet (δ_H_ 8.23) that were indicative of a protonated terminal amino group and a secondary amide functionality, respectively [7]. ^13^C NMR shifts and COSY correlations associated with the methylene signals of **7** enabled the assignment of an NH–(CH_2_)_3_–NH_2_ moiety, which was confirmed by HMBC correlations (Figure 3). This fragment was connected to the previously assigned moiety through a HMBC correlation from δ_H_ 3.25 to the amide carbonyl (δ_C_ 164.9), thus enabling the full chemical structure of ianthelliformisamine G (**7**) to be assigned.

**Table 2:** NMR data of ianthelliformisamines F (**6**) and G (**7**) in DMSO-*d*_6_.^a^

	Ianthelliformisamine F (**6**)			Ianthelliformisamine G (**7**)^b^	
			
position	δ_C_, type	δ_H_, mult. (*J* in Hz)		δ_C_, type	δ_H_, mult. (*J* in Hz)

1	134.4, C			134.3, C	
2	131.6, CH	7.88, s		131.6, CH	7.89, s
3	118.0, C			118.0, C	
4	153.9, C			153.9, C	
4-OCH_3_	60.6, CH_3_	3.81, s		60.6, CH_3_	3.82, s
5	118.0, C			118.0, C	
6	131.6, CH	7.88, s		131.6, CH	7.89, s
7	135.7, CH	7.32, d (15.8)		135.4, CH	7.35, d (15.8)
8	124.6, CH	6.65, d (15.8)		124.2, CH	6.65, d (15.8)
9	166.1, C			164.9, C	
9-NH_2_		7.19, brs			
		7.46, brs			
9-NH					8.23, t (5.9)
10				35.9, CH_2_	3.25, dt (5.9, 6.8)
11				27.5, CH_2_	1.73, tt (6.8, 7.5)
12				36.9, CH_2_	2.81, m
12-NH_2_					7.73, brs

^a^Spectra recorded at 25 °C (800 MHz for ^1^H NMR and 200 MHz for ^13^C NMR); ^b^isolated as a TFA salt.

Compounds **6** and **7** are both commercially available [16] but neither of these compounds has been fully characterised with only the total synthesis of **7** being reported in the literature [8]. Our study reports the first identification of **6** and **7** from a natural origin and the first full characterisation of these molecules using NMR, UV, and MS data.

**Scheme 1:**
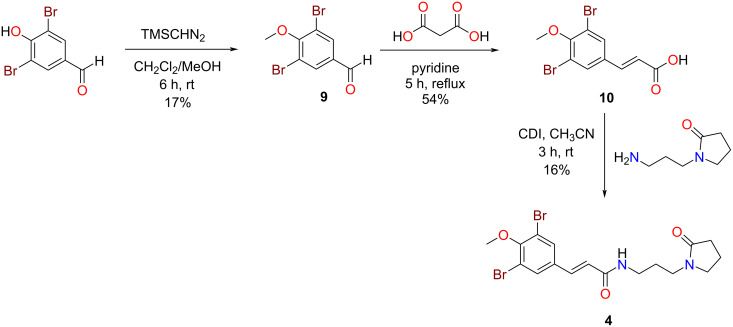
Total synthesis of ianthelliformisamine D (**4**).

Owing to the novelty of ianthelliformisamine D (**4**), we attempted the first total synthesis of this natural product (Scheme 1). In a similar, but modified manner to that reported by Gan et al. [19] we firstly methylated the commercially sourced 3,5-dibromo-4-hydroxybenzaldehyde using TMSCHN_2_ in MeOH/CH_2_Cl_2_ at room temperature (17% yield). Subjecting the methoxylated benzaldehyde intermediate **9** to a Doebner–Knoevenagel condensation with malonic acid and pyridine afforded the brominated cinnamic acid analogue **10** in 54% yield [19]. Amidation chemistry using carbonyldiimidazole (CDI) [18] and the commercially available primary amine, 1-(3-aminopropyl)pyrrolidin-2-one completed the total synthesis of the natural product in an overall yield of 1.5%. The NMR data comparison of the natural product and our synthetic compound was essentially identical.

Due to our interest in the identification of potential new leads against *Pseudomonas aeruginosa* and supported by numerous reports of favourable activity for ianthelliformisamines A–C [7,12] and their synthetic analogues [8–10], we investigated the planktonic and biofilm activity of the new natural products **4**–**7** in addition to the known metabolite, aplysterol (**8**) [13]. Our biological assessment of compounds **4**–**8** showed no inhibition of planktonic growth or biofilm formation for *P. aeruginosa* when screened at 50 µM. Previously reported antibacterial assessment of ianthelliformisamines A–C (**1**–**3**) and their synthetic analogues has generated structure–activity relationship data, leading to speculation over the moieties responsible for their antibiotic effects. A reduction in the number of amines in the polymeric chain and the absence of a primary amine was noted to decrease bioactivity by Xu et al. [7]. Research reported by Khan et al. in 2014 [9] also supported the important role that the polyamine chain length plays in antibacterial activity. Of note, increasing research providing structure–activity relationship analyses shows that polyamines (including spermine) are bacterial membrane disruptors and are beneficial in enhancing activity of known antibacterial agents [20–21]. The absence of polyamine chains in ianthelliformisamines D–G (**4**–**7**) could explain the loss of activity seen in our assessment against *P. aeruginosa*. The synthetic molecule of **7** has been tested by other researchers against Gram-negative bacteria including *P. aeruginosa* with reported MIC values of >200 µg/mL [8], which is consistent with the data we report in this paper.

Finally, and of note, our studies described here report the first isolation of aplysterol (**8**) from the genus *Suberea*. To date, there has been no evaluation of this compound’s antibiotic potential towards *P. aeruginosa*, including biofilm inhibition. Furthermore, this is the first report of any biological assessment of aplysterol (**8**) as a pure compound, since prior studies have only tested a mixture of **8** with 24,28-didehydroaplysterol, where it was found to inhibit DNA topoisomerase II-α (MIC = 50 µM) [22].

## Conclusion

In summary, we report here the discovery and full characterisation of four new natural products, ianthelliformisamines D–G from the marine sponge *Suberea ianthelliformis*. Furthermore, we describe the first total synthesis of ianthelliformisamine D which contains a rare *N*-(3-aminopropyl)-2-pyrrolidone moiety. Whilst testing of these natural products against *Pseudomonas aeruginosa* showed that none of them inhibited planktonic growth or biofilm formation at 50 µM, synthetic efforts has generated sufficient quantities of the novel compound ianthelliformisamine D that will enable additional biological profiling.

## Experimental

### General experimental procedures

Specific rotations were recorded using a JASCO P-1020 polarimeter. UV data was recorded on a JASCO V-650 UV–vis spectrophotometer. NMR spectra were recorded at 25 °C on a Bruker Avance III HD 800 MHz NMR spectrometer equipped with a cryoprobe. The ^1^H and ^13^C chemical shifts were referenced to solvent peaks for DMSO-*d*_6_ at δ_H_ 2.50 and δ_C_ 39.52, respectively. LRESIMS data was recorded on an Ultimate 3000 RS UHPLC coupled to a Thermo Fisher Scientific ISQEC single quadruple ESI mass spectrometer. HRESIMS data was acquired on a Bruker maXis II ETD ESI-qTOF. Alltech Davisil (30–40 µm, 60 Å) C_8_-bonded silica and Alltech Davisil (30–40 µm, 60 Å) diol-bonded silica were used for pre-adsorption work before RP- or NP-HPLC, respectively. The pre-adsorbed material was subsequently packed into an Alltech stainless steel guard cartridge (10 × 30 mm) then attached to a HPLC column prior to fractionation. A Waters 600 pump fitted with a Waters 996 photodiode array detector fitted with a Gilson 717-plus autosampler were used for RP-HPLC separations. A Thermo Fisher Scientific Dionex Ultimate 3000 UHPLC was used for NP-HPLC separations. Thermo Betasil phenyl-bonded silica (5 μm, 100 Å, 150 × 21.2 mm) and Phenomenex Luna C_18_ column (5 µm, 90–110 Å, 10 mm × 250 mm) were used for RP-HPLC separations. For NP-HPLC, a YMC diol-bonded silica (5 μm, 120 Å, 150 × 20 mm) column was used. The frozen marine sponge was dried using a Dynamic FD12 freeze dryer and ground using a Fritsch Universal Cutting Mill Pulverisette 19. The ground marine sponge was extracted at room temperature using an Edwards Instrument Company Bioline orbital shaker set to 200 rpm. Solvents were removed from extracts with a Büchi R-144 rotary evaporator and from HPLC fractions using a GeneVac XL4 centrifugal evaporator. All solvents used for chromatography, UV, MS, and [α]_D_ were Honeywell Burdick & Jackson or Lab-Scan HPLC grade. H_2_O was filtered using a Sartorius Stedium Arium Pro VF ultrapure water system. Reagents used for the total synthesis of Ianthelliformisamine D were purchased from either Sigma or Aaron Chemicals.

### Sponge material

The sponge sample was obtained from the NatureBank [23] biota library housed at the Institute for Biomedicine and Glycomics, Griffith University, Australia. A voucher specimen of *Suberea ianthelliformis* (NB6014998; phylum Porifera, class Demospongiae, order Verongida, family Aplysinellidae) has been previously lodged (G322255) at the Queensland Museum, South Brisbane, Queensland, Australia [7].

### Extraction and isolation

In a manner similar to that reported by Xu et al. [7], the freeze-dried and ground specimen of *S. ianthelliformis* (10 g) was extracted sequentially with *n*-hexane (250 mL, 2 h), CH_2_Cl_2_ (250 mL, 2 h), and MeOH (250 mL, 2 h; 250 mL, 16 h). The *n*-hexane extract was discarded (as it contained only highly lipophilic material) and the CH_2_Cl_2_ and MeOH extracts were combined to produce a brown gum (173.3 mg) that was pre-adsorbed to C_8_-bonded silica (≈1 g), packed into a stainless-steel guard cartridge, and subjected to phenyl semipreparative RP-HPLC separation. Isocratic conditions of 90% H_2_O (0.1% TFA)/10% MeOH (0.1% TFA) were initially employed for the first 10 min, then a linear gradient to 100% MeOH (0.1% TFA) was run over 40 min, followed by isocratic conditions of 100% MeOH (0.1% TFA) for a further 10 min, all at a flowrate of 9 mL/min; 60 fractions (60 × 1 min) were collected. This first HPLC fractionation afforded ianthelliformisamine B (**2**, 10.4 mg, *t*_R_ 36–37 min, 0.104% dry wt) and several other UV-active fractions that contained mixtures of polyamine-type alkaloids, which were combined for further isolation work. The following describes the purification of these fractions. Fractions 34–35 (5.0 mg) were purified by semipreparative phenyl RP-HPLC using isocratic conditions of 70% H_2_O (0.1% TFA)/30% MeOH (0.1% TFA) for the first 10 min, then a linear gradient to 10% H_2_O (0.1% TFA)/90% MeOH (0.1% TFA) was run over 50 min at a flowrate of 9 mL/min; 60 fractions (60 × 1 min) were collected and resulted in the purification of ianthelliformisamine A (**1**, 1.0 mg, *t*_R_ 16 min, 0.010% dry wt). Fractions 38–40 were combined (45.0 mg) and purified by semipreparative phenyl RP-HPLC using the same solvent gradient as above and afforded the new natural products, ianthelliformisamines G (**7**, 1.2 mg, *t*_R_ 19 min, 0.012% dry wt) and E (**5**, 1.5 mg, *t*_R_ 23 min, 0.015% dry wt). Fractions 41–42 were combined (22.4 mg) and purified by semipreparative RP-HPLC using the same solvent gradient and afforded the new natural products, ianthelliformisamines F (**6**, 1.2 mg, *t*_R_ 19 min, 0.012% dry wt) and D (**4**, 1.3 mg, *t*_R_ 41 min, 0.013% dry wt). Fractions 43–45 were combined (28.7 mg) and purified by semipreparative RP-HPLC using the same solvent gradient and afforded the known metabolite, ianthelliformisamine C (**3**, 1.8 mg, *t*_R_ 38 min, 0.018% dry wt). Fractions 46–60 (20.1 mg) was purified by semipreparative NP-HPLC. Isocratic conditions of 100% *n*-hexane were initially employed for the first 10 min, then a linear gradient to 80% *n*-hexane/20% iPrOH was run over 50 min at a flow rate of 9 mL/min; 60 fractions (60 × 1 min) were collected and resulted in the purification of the known sterol, aplysterol (**8**, 0.9 mg, *t*_R_ 12–13 min, 0.009% dry wt). The extraction and isolation process described above was repeated twice more (identical scale) to obtain sufficient quantities of the minor natural products for characterisation and biological assessment with similar recoveries obtained.

**Ianthelliformisamine D (4):** Stable brown gum; UV (MeOH) λ_max_, nm (log ε): 226 (4.01), 278 (3.86); ^1^H and ^13^C NMR data (DMSO-*d*_6_), see Table 1; LRESIMS (*m/z*): 459/461/463 [M + H]^+^; HRESIMS (*m/z*): [M + H]^+^ calcd for C_17_H_21_^79^Br_2_N_2_O_3_, 458.9913; found, 458.9917.

**Ianthelliformisamine E TFA salt (5):** Stable brown gum; UV (MeOH) λ_max_, nm (log ε): 229 (4.25), 279 (4.17); ^1^H and ^13^C NMR data (DMSO-*d*_6_), see Table 1; LRESIMS (*m/z*): 477/479/481 [M + H]^+^; HRESIMS (*m/z*): [M + H]^+^ calcd for C_17_H_23_^79^Br_2_N_2_O_4_, 477.0019; found, 477.0022.

**Ianthelliformisamine F (6):** Stable brown gum; UV (MeOH) λ_max_, nm (log ε): 229 (3.83), 280 (3.71); ^1^H and ^13^C NMR data (DMSO-*d*_6_), see Table 2; LRESIMS (*m/z*): 334/336/338 [M + H]^+^; HRESIMS (*m/z*): [M + Na]^+^ calcd for C_10_H_9_^79^Br_2_NO_2_Na, 355.8892; found, 355.8896.

**Ianthelliformisamine G TFA salt (7):** Stable brown gum; UV (MeOH) λ_max_, nm (log ε): 229 (3.63), 280 (3.57); ^1^H and ^13^C NMR data (DMSO-*d*_6_), see Table 2; LRESIMS (*m/z*): 391/393/395 [M + H]^+^; HRESIMS (*m/z*): [M + H]^+^ calcd for C_13_H_17_^79^Br_2_N_2_O_2_, 390.9651; found, 390.9647.

**Aplysterol (8):** Stable white powder; 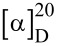 −46.7 (*c* 0.025, CHCl_3_), lit. 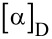 −25 (*c* not specified, CHCl_3_) [24]; HRESIMS (*m/z*): [M + Na]^+^ calcd for C_29_H_50_ONa, 437.3754; found, 437.3741.

#### Methylation of 3,5-dibromo-4-hydroxybenzaldehyde

3,5-Dibromo-4-hydroxybenzaldehyde (279.0 mg, 1.0 mmol) was dissolved in CH_2_Cl_2_/MeOH 1:1 (1 mL) then a solution of TMSCHN_2_ in hexanes (2.0 M, 1.5 mL, 11 mmol) was slowly added and the mixture stirred at room temperature for 6 h. The reaction crude was pre-adsorbed to C_8_-bonded silica, packed into a stainless steel guard cartridge then purified by Luna C_18_ semipreparative RP-HPLC using isocratic conditions of 50% H_2_O (0.1% TFA)/50% MeOH (0.1% TFA) for the first 5 min, then a linear gradient to 100% MeOH (0.1% TFA) was run over 20 min and held at 100% MeOH (0.1% TFA) for a further 5 min at a flowrate of 4 mL/min; 30 fractions (30 × 1 min) were collected and resulted in the purification of 3,5-dibromo-4-methoxybenzaldehyde (**9**, 50.1 mg, *t*_R_ 18–22 min, 17% yield) [19].

**3,5-Dibromo-4-methoxybenzaldehyde (9):** White powder; ^1^H NMR (DMSO-*d*_6_, 800 MHz) δ_H_ 9.89 (s, 1H, H-7), 8.17 (s, 2H, H-2, H-6), 3.88 (s, 3H, 4-OCH_3_); ^13^C NMR (DMSO-*d*_6_, 200 MHz) δ_C_ 190.1 (C-7), 158.1 (C-4), 134.5 (C-1), 133.8 (2C, C-2, C-6), 118.6 (2C, C-3, C-5), 60.8 (4-OCH_3_); LRESIMS (*m/z*): 293/295/297 [M + H]^+^.

#### Doebner–Knoevenagel condensation of 3,5-dibromo-4-methoxybenzaldehyde with malonic acid

3,5-Dibromo-4-methoxybenzaldehyde (**9**, 80.0 mg, 0.27 mmol), malonic acid (56.0 mg, 0.54 mmol) and dry pyridine (1 mL) were refluxed at 100 °C for 5 h. The reaction crude was pre-adsorbed to C_8_-bonded silica, packed into a stainless steel guard cartridge then purified by Betasil semipreparative RP-HPLC using isocratic conditions of 90% H_2_O (0.1% TFA)/10% MeOH (0.1% TFA) that were initially employed for the first 10 min, then a linear gradient to 100% MeOH (0.1% TFA) was run over 40 min, followed by isocratic conditions of 100% MeOH (0.1% TFA) for a further 10 min, all at a flowrate of 9 mL/min; 60 fractions (60 × 1 min) were collected resulting in the purification of (*E*)-3-(3,5-dibromo-4-methoxyphenyl)acrylic acid (**10**, 49.0 mg, *t*_R_ 46–48 min, 54% yield) [19].

**(*E*)-3-(3,5-Dibromo-4-methoxyphenyl)acrylic acid (10):** White powder; ^1^H NMR (DMSO-*d*_6_, 800 MHz) δ_H_ 8.06 (s, 2H, H-2, H-6), 7.50 (d, *J* = 16.0 Hz, 1H, H-7), 6.62 (d, *J* = 16.0 Hz, 1H, H-8), 3.82 (s, 3H, 4-OCH_3_); ^13^C NMR (DMSO-*d*_6_, 200 MHz) δ_C_ 167.3 (C-9), 154.4 (C-4), 140.5 (C-7), 133.7 (C-1), 132.4 (2C, C-2, C-6), 121.5 (C-8), 118.0 (2C, C-3, C-5) 60.6 (4-OCH_3_) ; LRESIMS (*m/z*): 333/335/337 [M − H]^−^.

#### Synthesis of Ianthelliformisamine D

(*E*)-3-(3,5-Dibromo-4-methoxyphenyl)acrylic acid (**10**, 49.0 mg, 0.15 mmol) and 1,1′-carbonyldiimadazole (24.0 mg, 0.15 mmol) was added to a reaction vial with dry CH_3_CN (400 µL) and stirred at room temperature for 10 min. 1-(3-Aminopropyl)pyrrolidin-2-one (100 µL, 0.60 mmol) was then added dropwise and the resulting solution was stirred at room temperature for 3 h. The reaction crude was pre-adsorbed to C_8_-bonded silica, packed into a stainless steel guard cartridge then purified by Luna semipreparative RP-HPLC using isocratic conditions of 50% H_2_O (0.1% TFA)/50% MeOH (0.1% TFA) for the first 5 min, then a linear gradient to 100% MeOH (0.1% TFA) was run over 20 min and held at 100% MeOH (0.1% TFA) for a further 5 minutes at a flowrate of 4 mL/min; 30 fractions (30 × 1 min) were collected and resulted in the purification of synthetic Ianthelliformisamine D (**4**, 11.1 mg, *t*_R_ 21 min, 16% yield). The ^1^H and ^13^C NMR spectra of the synthetic compound matched that of the natural product.

**Synthetic Ianthelliformisamine D (4):** Clear film: ^1^H NMR (DMSO-*d*_6_, 800 MHz) δ_H_ 8.04 (t, *J* = 5.7 Hz, 1H, 9-NH), 7.88 (s, 2H, H-2, H-6), 7.33 (d, *J* = 15.7 Hz, 1H, H-7), 6.66 (d, *J* = 15.7 Hz, 1H, H-8), 3.81 (s, 3H, 4-OCH_3_), 3.33 (t, *J* = 7.1 Hz, 2H, H-13), 3.20 (t, *J* = 7.2 Hz, 2H, H-12), 3.14 (dt, *J* = 5.7, 6.8 Hz, 2H, H-10), 2.21 (t, *J* = 8.1 Hz, 2H, H-15), 1.91 (tt, *J* = 7.1, 8.1 Hz, 2H, H-14), 1.63 (tt, *J* = 6.8, 7.2 Hz, 2H, H-11); ^13^C NMR (DMSO-*d*_6_, 200 MHz) δ_C_ 173.9 (C-16), 164.4 (C-9), 153.9 (C-4), 135.2 (C-7), 134.5 (C-1), 131.6 (2C, C-2, C-6), 124.5 (C-8), 118.0 (2C, C-3, C-5) 60.5 (4-OCH_3_), 46.4 (C-13), 39.6 (C-12), 36.5 (C-10), 30.5 (C-15), 26.9 (C-11), 17.5 (C-14); LRESIMS (*m/z*): 459/461/463 [M + H]^+^.

### Bacterial strains and media

Wild-type *Pseudomonas aeruginosa* strain PAO1 (prototrophic wild-type) [25] was used in this study. Bacteria were grown in Luria–Bertani (LB) medium (10 g/L tryptone, 10 g/L sodium chloride and 5 g/L yeast extract).

### Biofilm inhibition assay

The overnight cultures at 37 °C in LB medium were washed once with sterile saline 0.9% (w/v) and adjusted to an OD_600_ of 0.05. Then, 1% inoculum was transferred into fresh LB medium. Following the incubation at 37 °C, 200 rpm for 5–6 h to reach the mid-log phase, the cells were washed once with sterile saline 0.9% (w/v) and diluted to an OD_600_ of 0.01. Test compounds (15 µL) were loaded prior to the addition of bacteria (135 µL) into 96-well plates. The plates were incubated for 24 h at 37 °C in static conditions. The effects of compounds on bacterial growth and the viability of biofilm bacteria was determined by the OD_600_ and resazurin (RSZ) metabolic assay, respectively. The final concentration of DMSO in the assays was 1% (v/v). The negative controls consisted of inoculum and 1% DMSO. The antibiotic tobramycin (Sigma-Aldrich; 16 µg/mL) was used as a positive control. The initial OD_600_ and final OD_600_ were read before incubation at 37 °C and after 24 h of incubation, respectively, followed by the assessment of biofilm viability by resazurin metabolic assay. The experiments were carried out with three technical replicates and three biological replicates. The assay was performed as previously described [26]. The cultures were withdrawn, and the plates were washed twice with sterile water. Then, 50 µL of diluted RSZ solution (0.2% w/v) in LB medium was added into each well followed by incubation at 37 °C for 5 h. A microplate reader (iD5 Multi-Mode) was used to measure the fluorescence intensity (excitation 530 nm, emission 590 nm).

## Supporting Information

File 1 NMR data tables for compounds **4**–**7**, 1D and 2D NMR spectra of compounds **4**–**7**, ^1^H NMR spectra of natural products **1**–**3** and **8** and ^1^H and ^13^C NMR spectra of synthetic compounds **4, 9**, and **10**.

## Data Availability

Data generated and analyzed during this study is available from the corresponding author upon reasonable request.
