# Supplementary material for: Discovery of ianthelliformisamines D–G from the sponge Suberea ianthelliformis and the total synthesis of ianthelliformisamine D
Source: Beilstein J Org Chem. 2024 Dec 9;20:3205–14. doi: 10.3762/bjoc.20.266 (PMC11650616; doi:10.3762/bjoc.20.266)
Supplement: File 1 — NMR data tables for compounds 4–7, 1D and 2D NMR spectra of compounds 4–7, 1H NMR spectra of natural products 1–3 and 8 and 1H and 13C NMR spectra of synthetic compounds 4, 9, and 10. [file Beilstein_J_Org_Chem-20-3205-s001.pdf]

BEILSTEIN JOURNAL OF ORGANIC CHEMISTRY

Supporting Information

for

Discovery of ianthelliformisamines D–G from the sponge *Suberea ianthelliformis* and the total synthesis of ianthelliformisamine D

Sasha Hayes, Yaoying Lu, Bernd H. A. Rehm and Rohan A. Davis

Beilstein J. Org. Chem. **2024,** *20,* 3205–3214. doi:10.3762/bjoc.20.266

NMR data tables for compounds 4–7, 1D and 2D NMR spectra of compounds 4–7, ^{1}H NMR spectra of natural products 1–3 and 8 and ^{1}H and ^{13}C NMR spectra of synthetic compounds 4, 9, and 10

License and Terms: This is a supporting information file under the terms of the Creative Commons Attribution License (https://creativecommons.org/licenses/by/4.0). Please note that the reuse, redistribution and reproduction in particular requires that the author(s) and source are credited and that individual graphics may be subject to special legal provisions.
The license is subject to the *Beilstein Journal of Organic Chemistry* terms and conditions: (https://www.beilstein-journals.org/bjoc/terms)

Table of contents:

**Table S1**	NMR data table for ianthelliformisamine D (**4**) in DMSO-d_6
**Figure S1**	^{1}H NMR (800 MHz) spectrum of ianthelliformisamine D (**4**) in DMSO-d_6
**Figure S2**	^{13}C NMR (200 MHz) spectrum of ianthelliformisamine D (**4**) in DMSO-d_6
**Figure S3**	HSQC spectrum of ianthelliformisamine D (**4**) in DMSO-d_6
**Figure S4**	HMBC spectrum of ianthelliformisamine D (**4**) in DMSO-d_6
**Figure S5**	COSY spectrum of ianthelliformisamine D (**4**) in DMSO-d_6
**Figure S6**	ROESY spectrum of ianthelliformisamine D (**4**) in DMSO-d_6
**Table S2**	NMR data table for ianthelliformisamine E (**5**) in DMSO-d_6
**Figure S7**	^{1}H NMR (800 MHz) spectrum of ianthelliformisamine E (**5**) in DMSO-d_6
**Figure S8**	^{13}C NMR (200 MHz) spectrum of ianthelliformisamine E (**5**) in DMSO-d_6
**Figure S9**	HSQC spectrum of ianthelliformisamine E (**5**) in DMSO-d_6
**Figure S10**	HMBC spectrum of ianthelliformisamine E (**5**) in DMSO-d_6
**Figure S11**	COSY spectrum of ianthelliformisamine E (**5**) in DMSO-d_6
**Figure S12**	ROESY spectrum of ianthelliformisamine E (**5**) in DMSO-d_6
**Table S3**	NMR data table for ianthelliformisamine F (**6**) in DMSO-d_6
**Figure S13**	^{1}H NMR (800 MHz) spectrum of ianthelliformisamine F (**6**) in DMSO-d_6
**Figure S14**	^{13}C NMR (200 MHz) spectrum of ianthelliformisamine F (**6**) in DMSO-d_6
**Figure S15**	HSQC spectrum of ianthelliformisamine F (**6**) in DMSO-d_6
**Figure S16**	HMBC spectrum of ianthelliformisamine F (**6**) in DMSO-d_6
**Figure S17**	COSY spectrum of ianthelliformisamine F (**6**) in DMSO-d_6
**Table S4**	NMR data table for ianthelliformisamine G (**7**) in DMSO-d_6
**Figure S18**	^{1}H NMR (800 MHz) spectrum of ianthelliformisamine G (**7**) in DMSO-d_6
**Figure S19**	^{13}C NMR (200 MHz) spectrum of ianthelliformisamine G (**7**) in DMSO-d_6

**Figure S20**	HSQC spectrum of ianthelliformisamine G (**7**) in DMSO-d_6
**Figure S21**	HMBC spectrum of ianthelliformisamine G (**7**) in DMSO-d_6
**Figure S22**	COSY spectrum of ianthelliformisamine G (**7**) in DMSO-d_6
**Figure S23**	^{1}H NMR (800 MHz) spectrum of aplysterol (**8**) in DMSO-d_6
**Figure S24**	^{13}C NMR (200 MHz) spectrum of aplysterol (**8**) in DMSO-d_6
**Figure S25**	^{1}H NMR (800 MHz) spectrum of ianthelliformisamine A (**1**) in DMSO-d_6
**Figure S26**	^{1}H NMR (800 MHz) spectrum of ianthelliformisamine B (**2**) in DMSO-d_6
**Figure S27**	^{1}H NMR (800 MHz) spectrum of ianthelliformisamine C (**3**) in DMSO-d_6
**Figure S28**	^{1}H NMR (800 MHz) spectrum of 3,5-dibromo-4-methoxybenzaldehyde (**9**) in DMSO-d_6
**Figure S29**	^{13}C NMR (200 MHz) spectrum of 3,5-dibromo-4-methoxybenzaldehyde (**9**) in DMSO-d_6
**Figure S30**	^{1}H NMR (800 MHz) spectrum of (*E*)-3-(3,5-dibromo-4-methoxyphenyl)acrylic acid (**10**) in DMSO-d_6
**Figure S31**	^{13}C NMR (200 MHz) spectrum of (*E*)-3-(3,5-dibromo-4-methoxyphenyl)acrylic acid (**10**) in DMSO-d_6
**Figure S32**	^{1}H NMR (800 MHz) spectrum of synthetic ianthelliformisamine D (**4**) in DMSO-d_6
**Figure S33**	^{13}C NMR (200 MHz) spectrum of synthetic ianthelliformisamine D (**4**) in DMSO-d_6
**Figure S34**	Stacked ^{1}H NMR (800 MHz) spectra of natural product (a) and synthetic (b), ianthelliformisamine D (**4**) in DMSO-d_6
**Figure S35**	Stacked ^{13}C NMR (200 MHz) spectra of natural product (a) and synthetic (b), ianthelliformisamine D (**4**) in DMSO-d_6

Table S1: NMR data table for ianthelliformisamine D (**4**) in DMSO-d_6[a]

position	δ_C, type	δ_H, mult. (J in Hz)	COSY	HMBC	ROESY
1	134.5, C				
2	131.6, CH	7.89, s		3, 4, 6, 7	7, 8
3	118.0, C				
4	153.9, C				
4-OCH_3	60.6, CH_3	3.81, s		4	
5	118.0, C				
6	131.6, CH	7.89, s		2, 4, 5, 7	7, 8
7	135.2, CH	7.33, d (15.8)	8	1^w, 2, 6, 8^w, 9	2, 6
8	124.5, CH	6.66, d (15.8)	7	1, 9	2, 6, 9-NH
9	164.4, C				
9-NH		8.04, t (5.7)	10	9, 10^w	8, 10^w
10	36.5, CH_2	3.14 dt (5.7, 6.5)	9-NH, 11	9, 11^w, 12	9-NH, 11, 12
11	27.0, CH_2	1.63, m	10, 12	10^w, 12	10, 12
12	39.7, CH_2	3.20, t (7.2)	11	10, 11^w, 13^w	10, 11
13	46.4, CH_2	3.33, m	14	12^w, 14^w, 15	14, 15
14	17.5, CH_2	1.91, m	13, 15	13, 15^w, 16	13, 15
15	30.5, CH_2	2.21, t (8.0)	14	13, 14, 16	13, 14
16	173.9				

[a]Spectra recorded at 25 °C (800 MHz for ^1H NMR and 200 MHz for ^{13}C NMR); [w]Weak correlation.

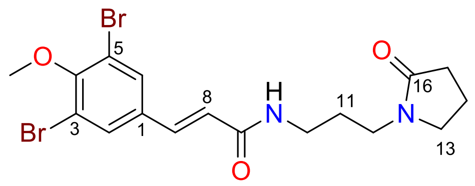

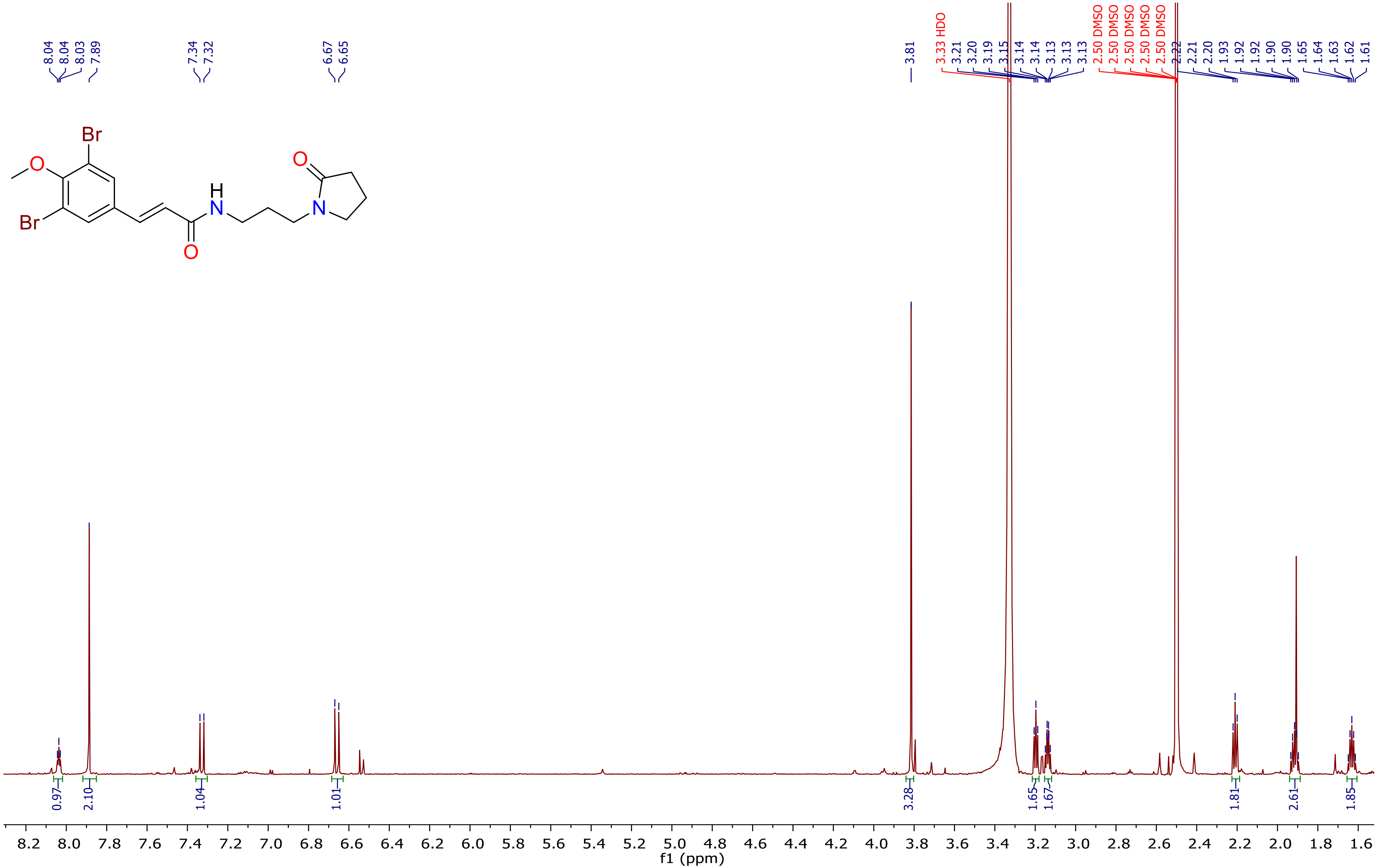

Figure S1: ^{1}H NMR (800 MHz) spectrum of ianthelliformisamine D (**4**) in DMSO-d_6

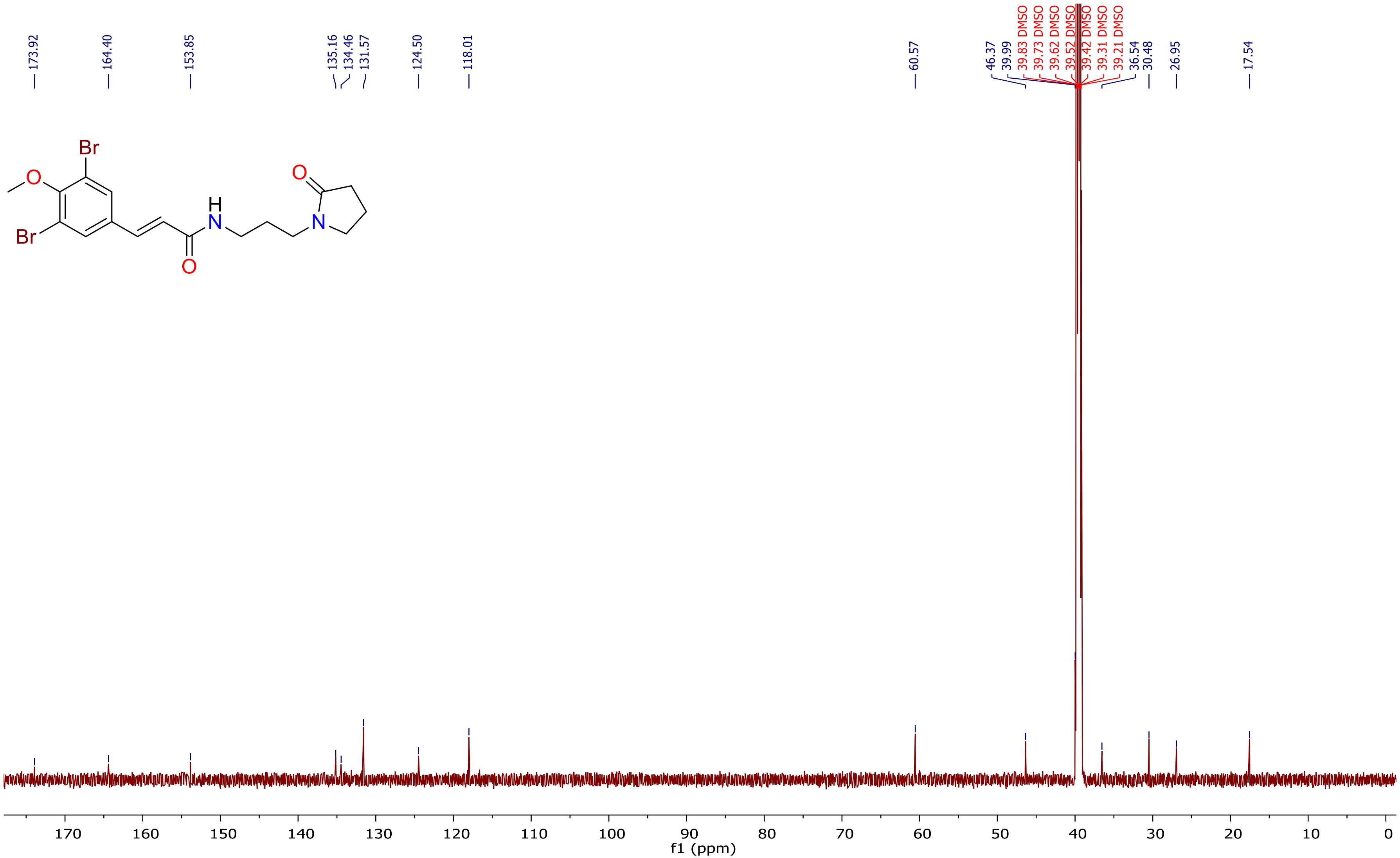

Figure S2: ^{13}C NMR (200 MHz) spectrum of ianthelliformisamine D (**4**) in DMSO-d_6

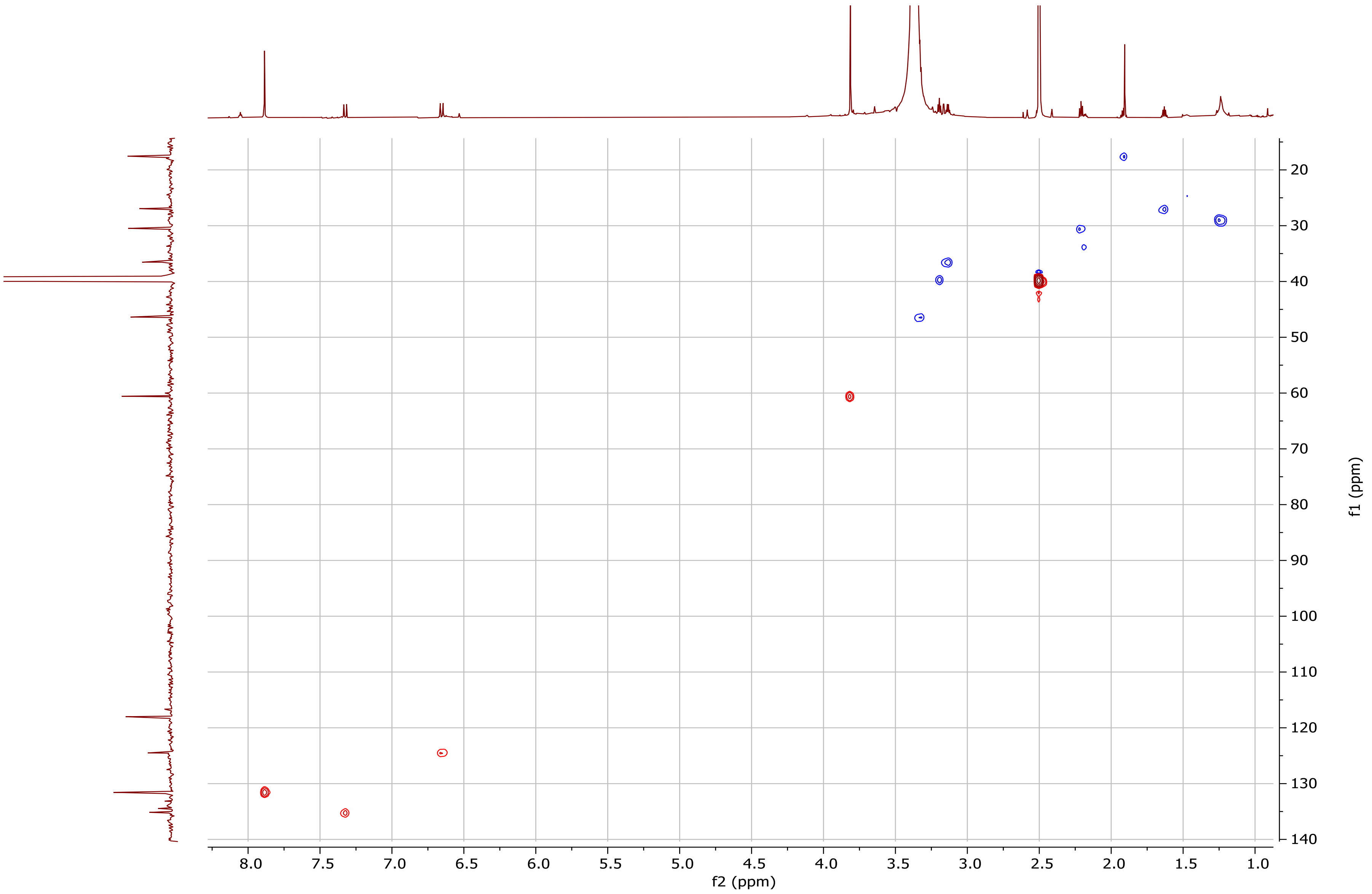

Figure S3: HSQC spectrum of ianthelliformisamine D (**4**) in DMSO-d_6

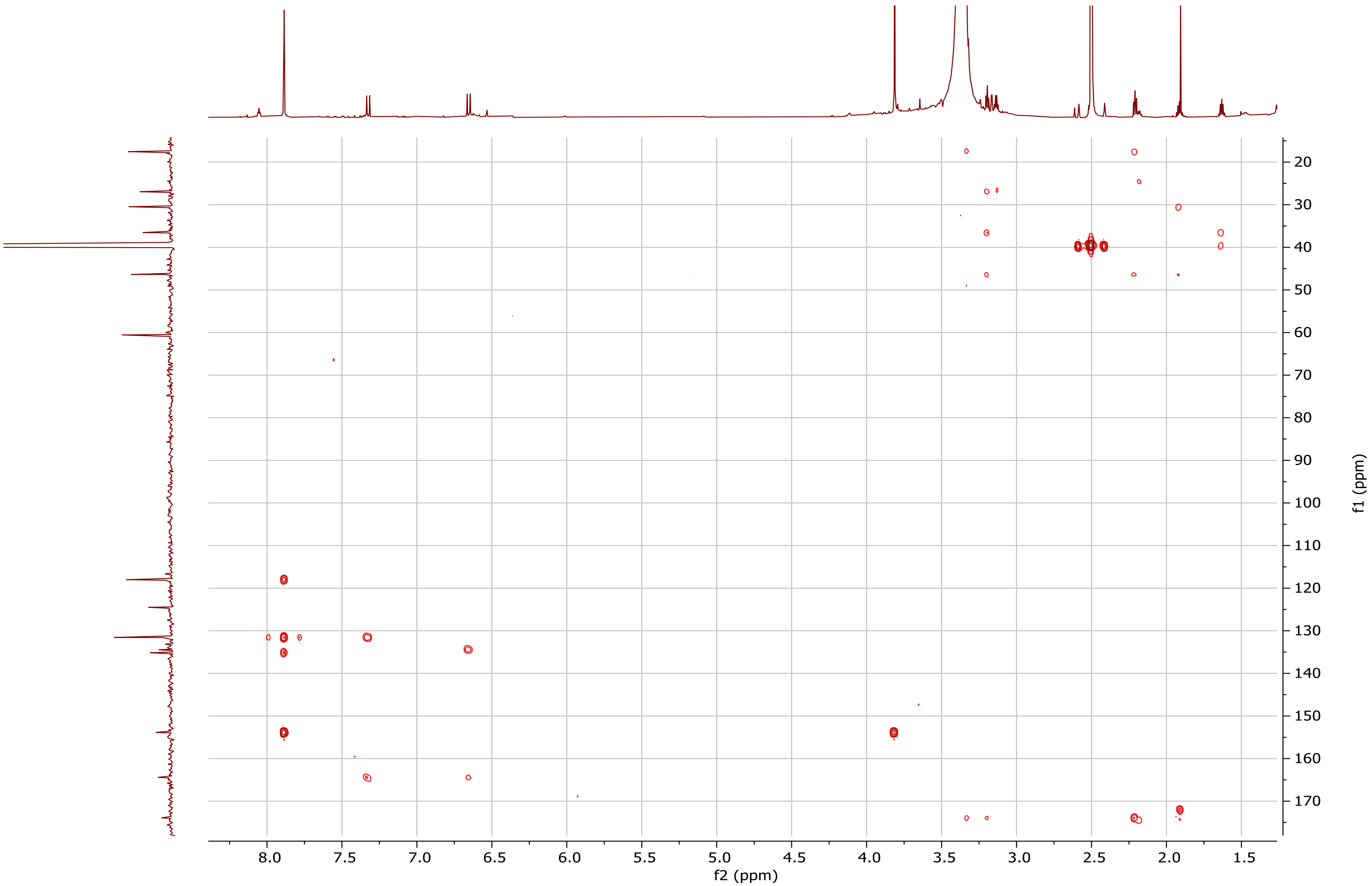

Figure S4: HMBC spectrum of ianthelliformisamine D (**4**) in DMSO-d_6

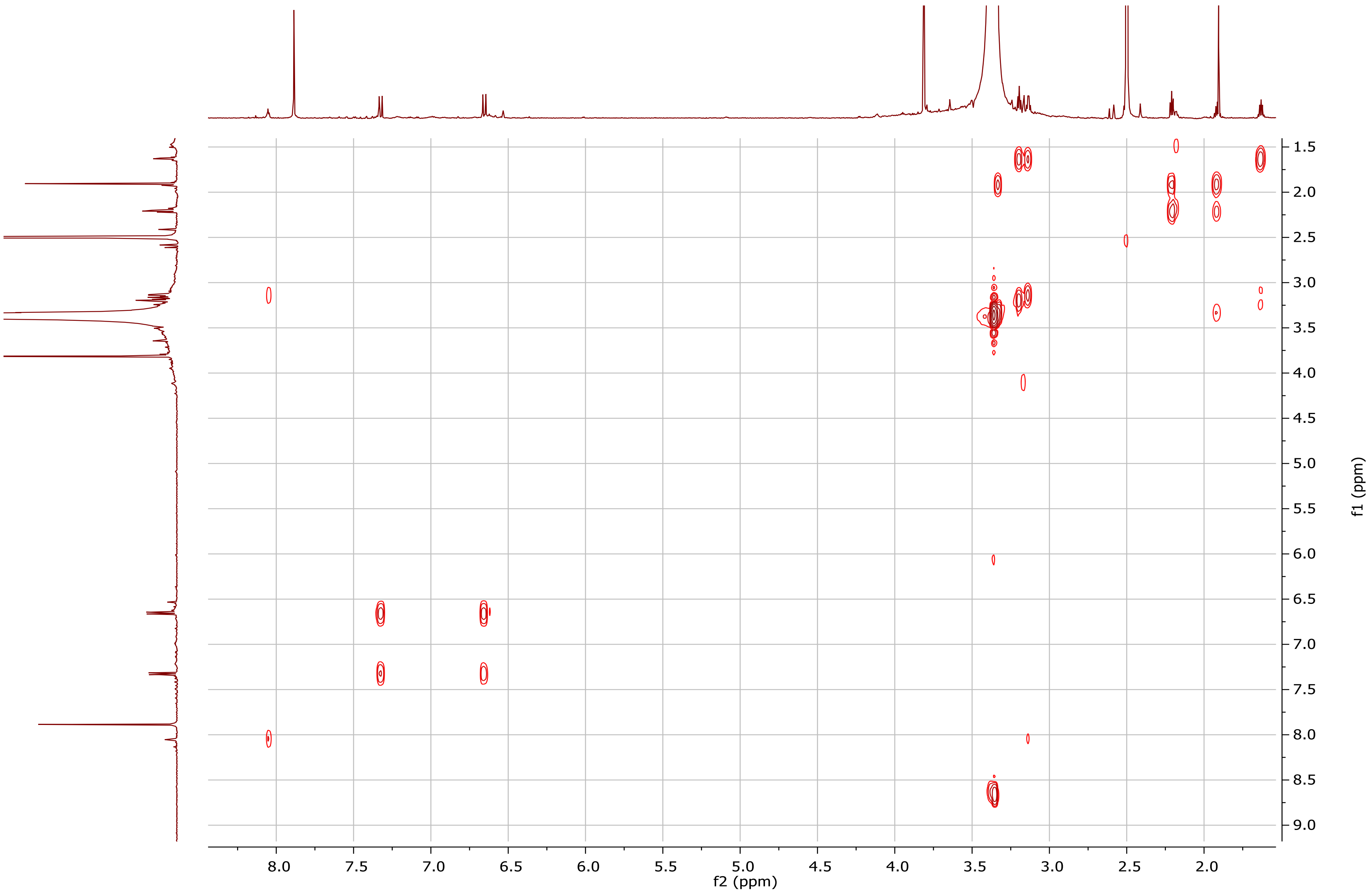

Figure S5: COSY spectrum of ianthelliformisamine D (**4**) in DMSO-d_6

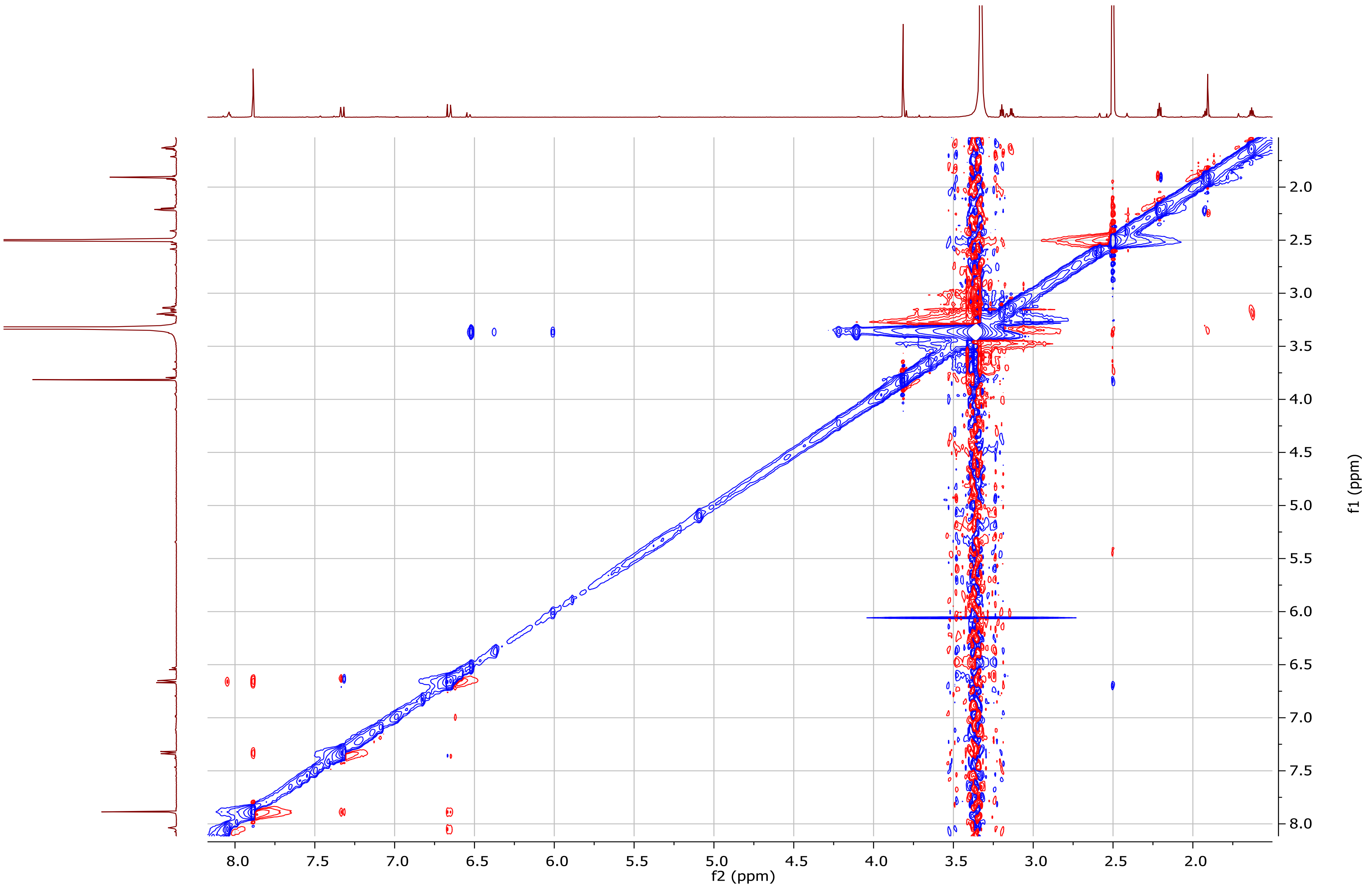

Figure S6: ROESY spectrum of ianthelliformisamine D (**4**) in DMSO-d_6

Table S2: NMR data table for ianthelliformisamine E (**5**) in DMSO-d_6[a, b]

position	δ_C, type	δ_H, mult. (J in Hz)	COSY	HMBC	ROESY
1	134.3, C				
2	131.6, CH	7.89, s		3, 4, 6, 7	7, 8
3	118.0, C				
4	153.9, C				
4-OCH$_3$	60.6, CH$_3$	3.82, s		4	
5	118.0, C				
6	131.6, CH	7.89, s		2, 4, 5, 7	7, 8
7	135.5, CH	7.36, d (15.8)	8	1[w], 2, 6, 8[w], 9	
8	124.1, CH	6.65, d (15.8)	7	1, 9	
9	164.9, C				
9-NH		8.23, t (5.7)	10	9, 10[w]	8, 10
10	35.9, CH$_2$	3.25 dt (5.7, 6.5)	9-NH, 11	9, 11[w], 12	11, 12
11	26.1, CH$_2$	1.77, m	10, 12	10[w], 12	10, 12
12	44.8, CH$_2$	2.92, m	11, 12-NH	10, 11[w], 13[w]	10, 11
12-NH		8.35, brs	12, 13		
13	46.3, CH$_2$	2.93, m	12-NH, 14	12[w], 14[w], 15	14, 15
14	21.1, CH$_2$	1.79, m	13, 15	13, 15[w], 16	13, 15
15	30.4, CH$_2$	2.36, t (7.3)	14	13, 14, 16	13, 14
16	173.6, C				
16-OH		[c]			

[a]Spectra recorded at 25 °C (800 MHz for ^{1}H NMR and 200 MHz for ^{13}C NMR); [b]Isolated as a TFA salt; [c] Not observed; [w]Weak correlation.

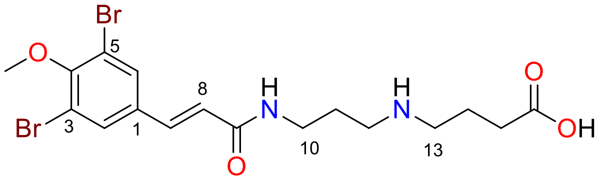

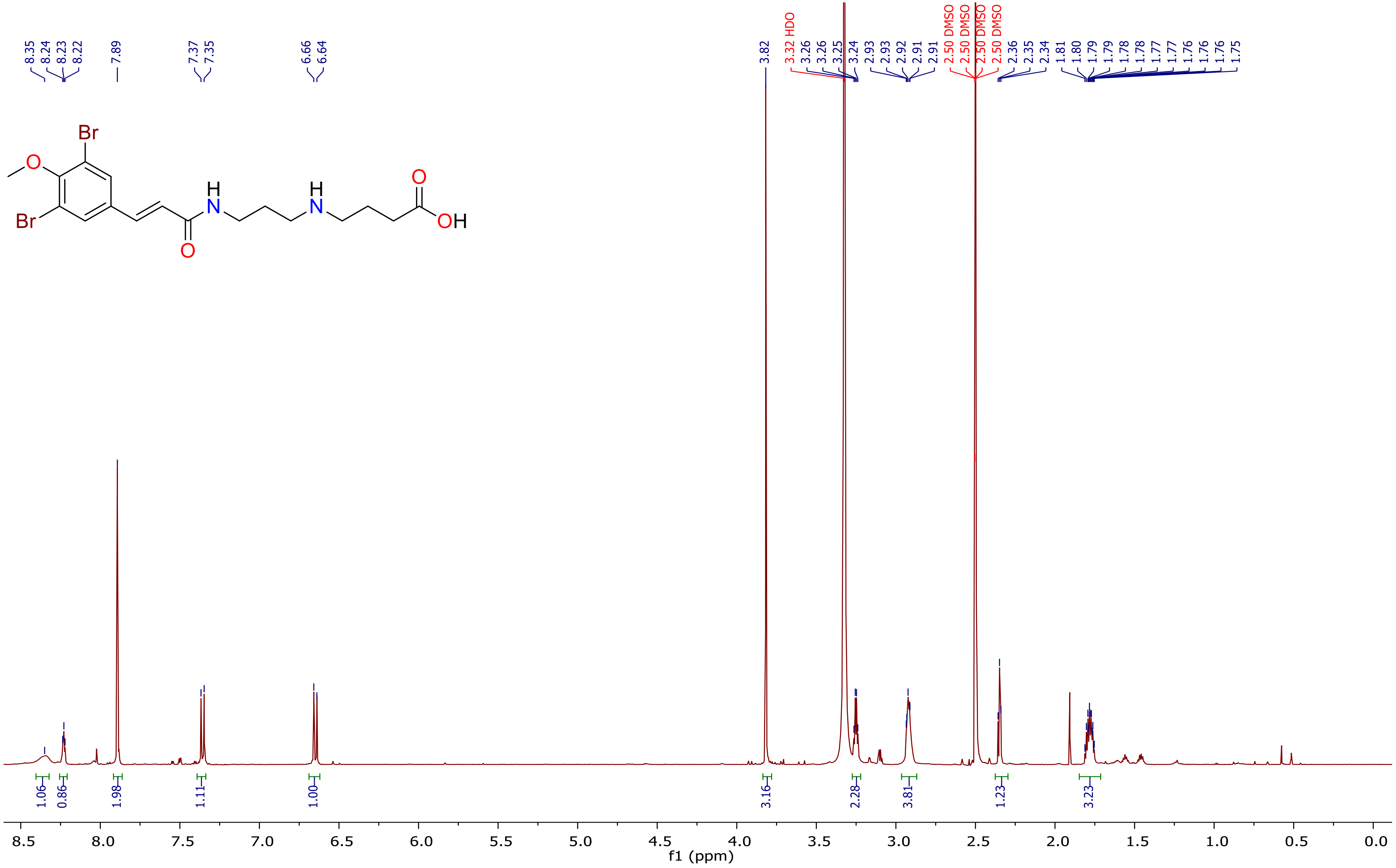

Figure S7: ^{1}H NMR (800 MHz) spectrum of ianthelliformisamine E (**5**) in DMSO-d_6

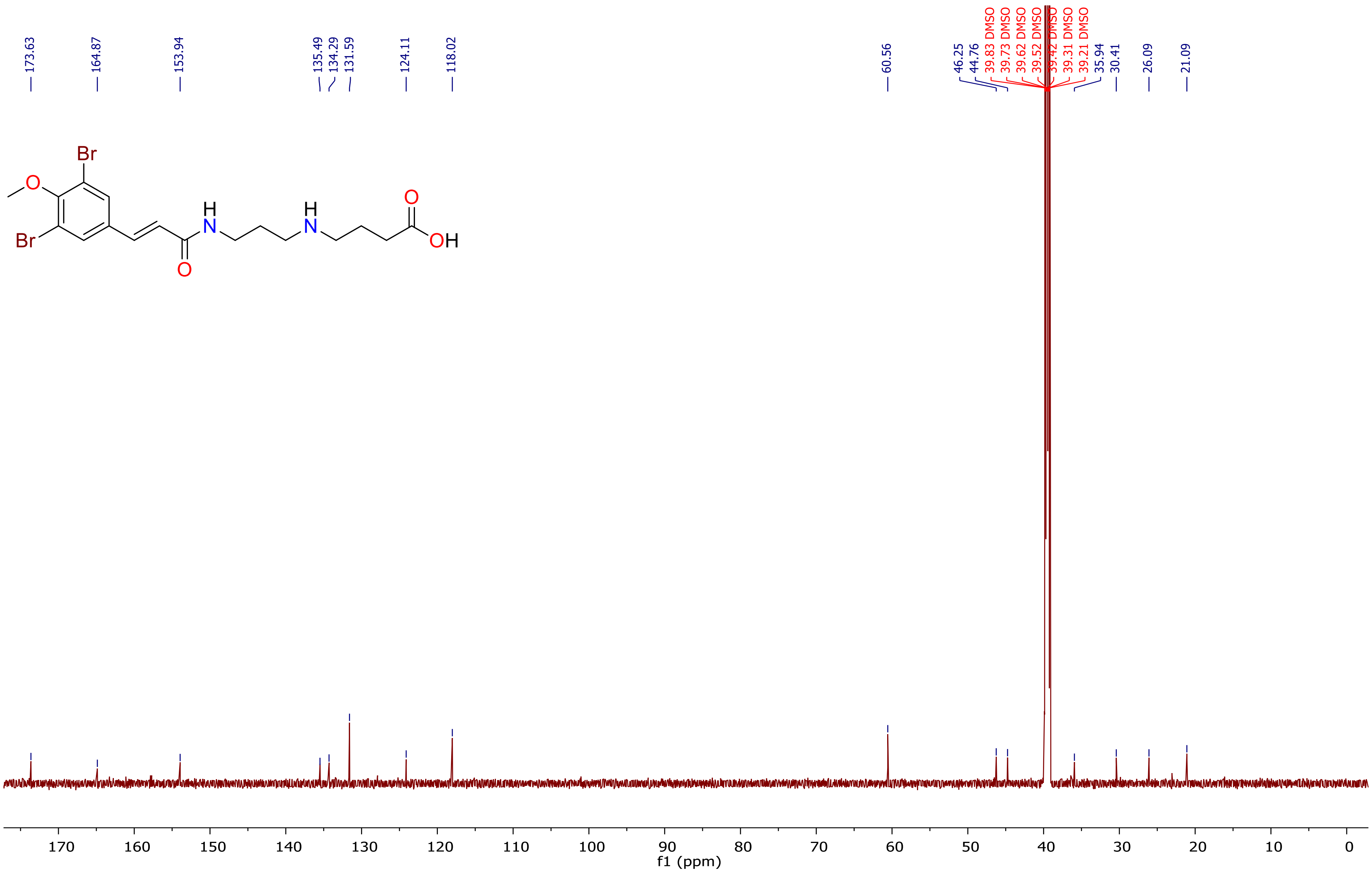

Figure S8: ^{13}C NMR (200 MHz) spectrum of ianthelliformisamine E (**5**) in DMSO-d_6

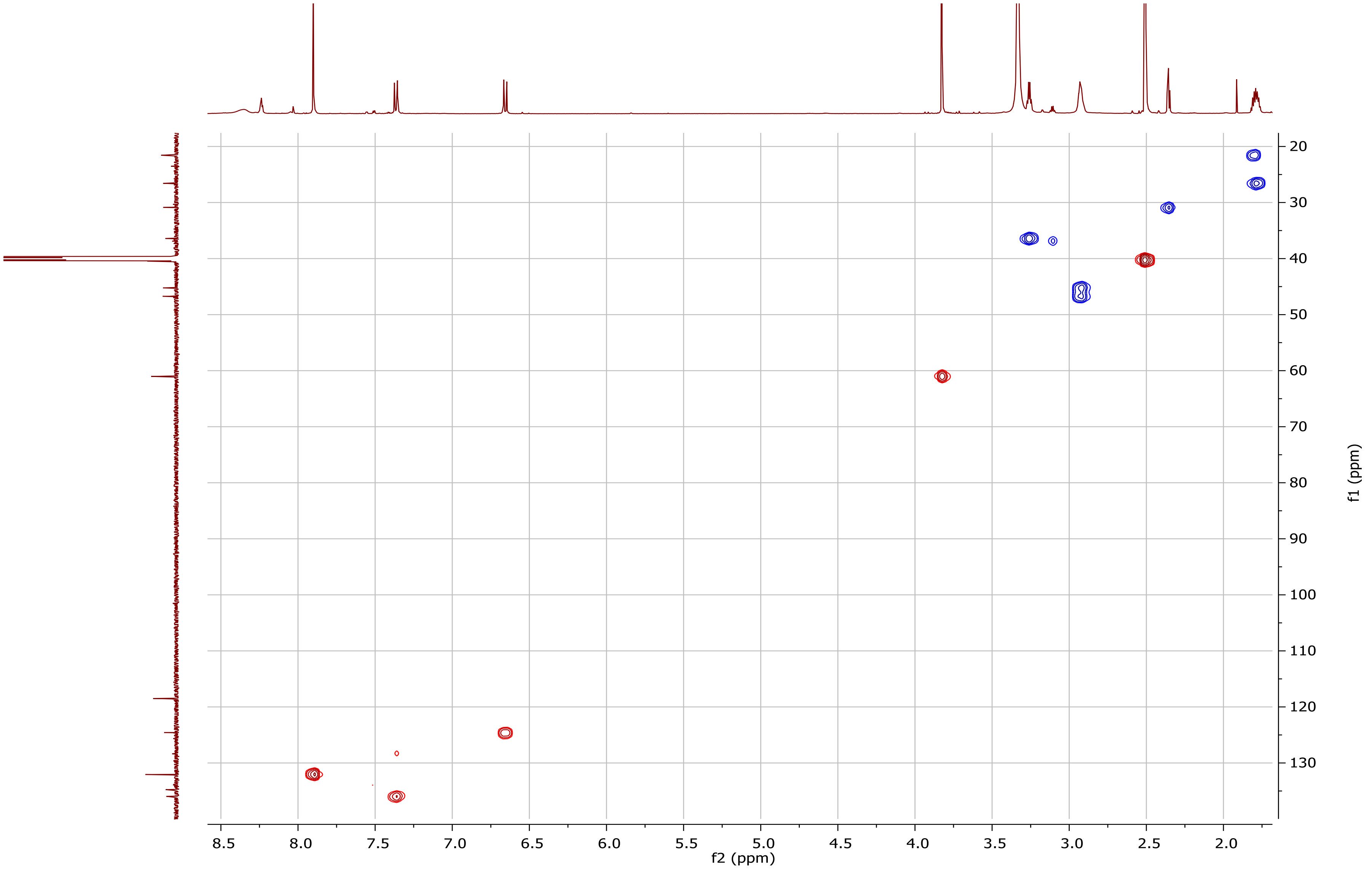

Figure S9: HSQC spectrum of ianthelliformisamine E (**5**) in DMSO-d_6

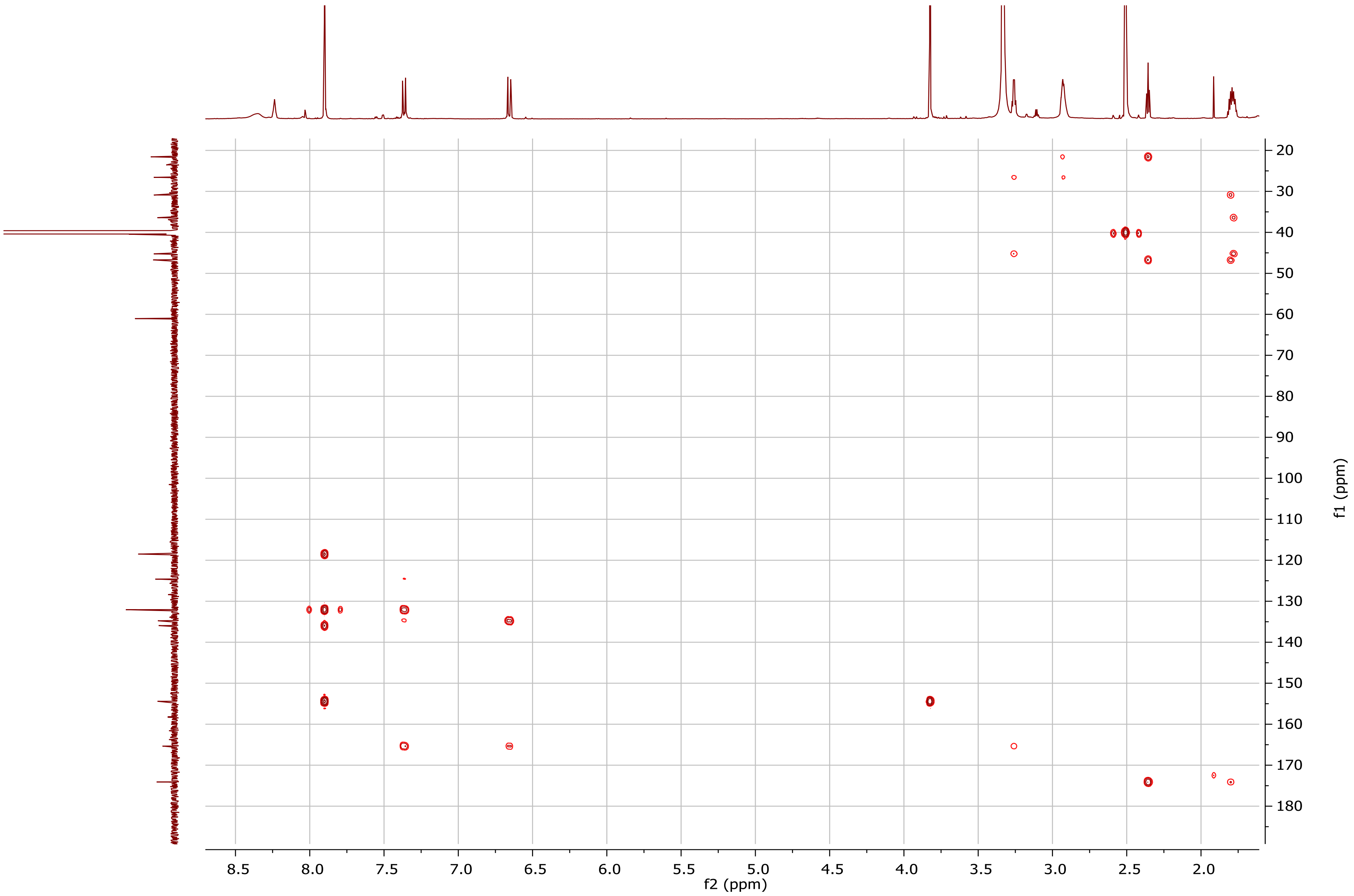

Figure S10: HMBC spectrum of ianthelliformisamine E (**5**) in DMSO-d_6

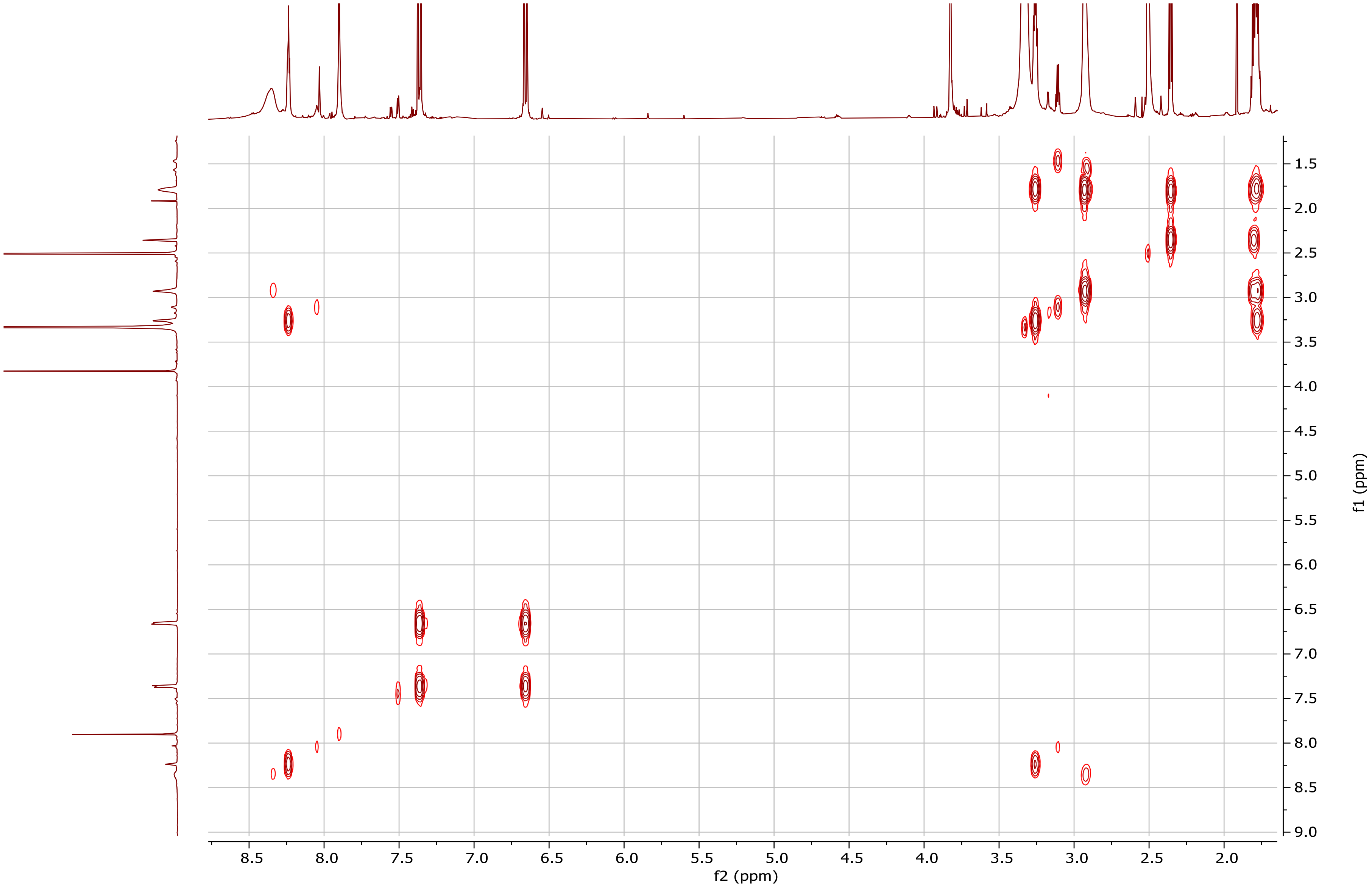

Figure S11: COSY spectrum of ianthelliformisamine E (**5**) in DMSO-d_6

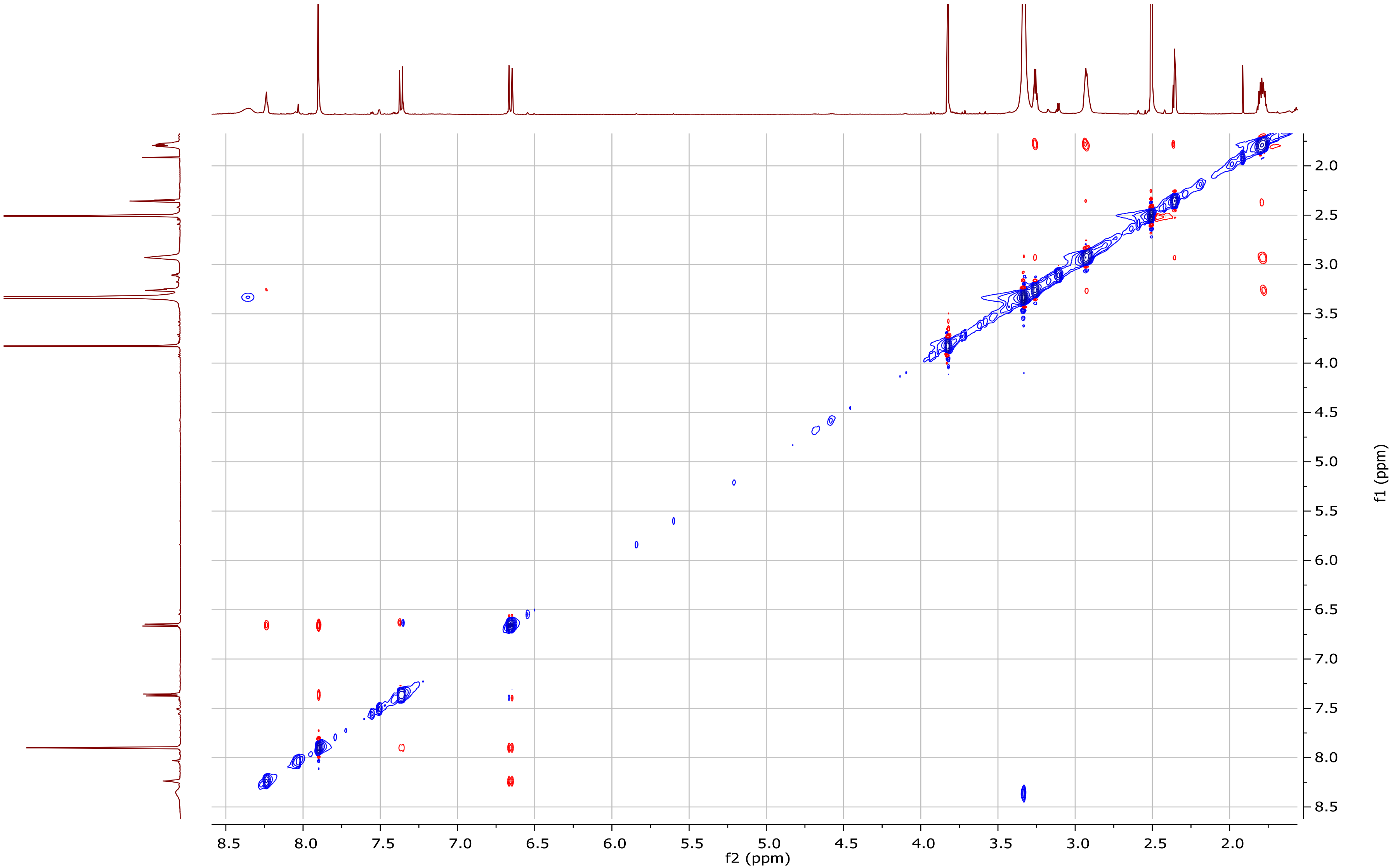

Figure S12: ROESY spectrum of ianthelliformisamine E (**5**) in DMSO-d_6

Table S3: NMR data table for ianthelliformisamine F (**6**) in DMSO-d_6[a]

position	δ_C, type	δ_H, mult. (J in Hz)	COSY	HMBC
1	134.4, C			
2	131.6, CH	7.88, s		1^w, 3, 4, 6, 7
3	118.0, C			
4	153.9, C			
4-OCH_3	60.6, CH_3	3.81, s		4
5	118.0, C			
6	131.6, CH	7.88, s		1^w, 2, 4, 5, 7
7	135.7, CH	7.32, d (15.8)	8	1, 2, 6, 8^w, 9
8	124.6, CH	6.65, d (15.8)	7	1, 7, 9
9	166.1, C			
9-NH_2		7.19, brs	9-NH_2	8
		7.46, brs	9-NH_2	

[a]Spectra recorded at 25 °C (800 MHz for ^{1}H NMR and 200 MHz for ^{13}C NMR).

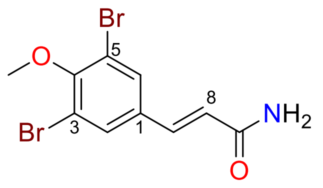

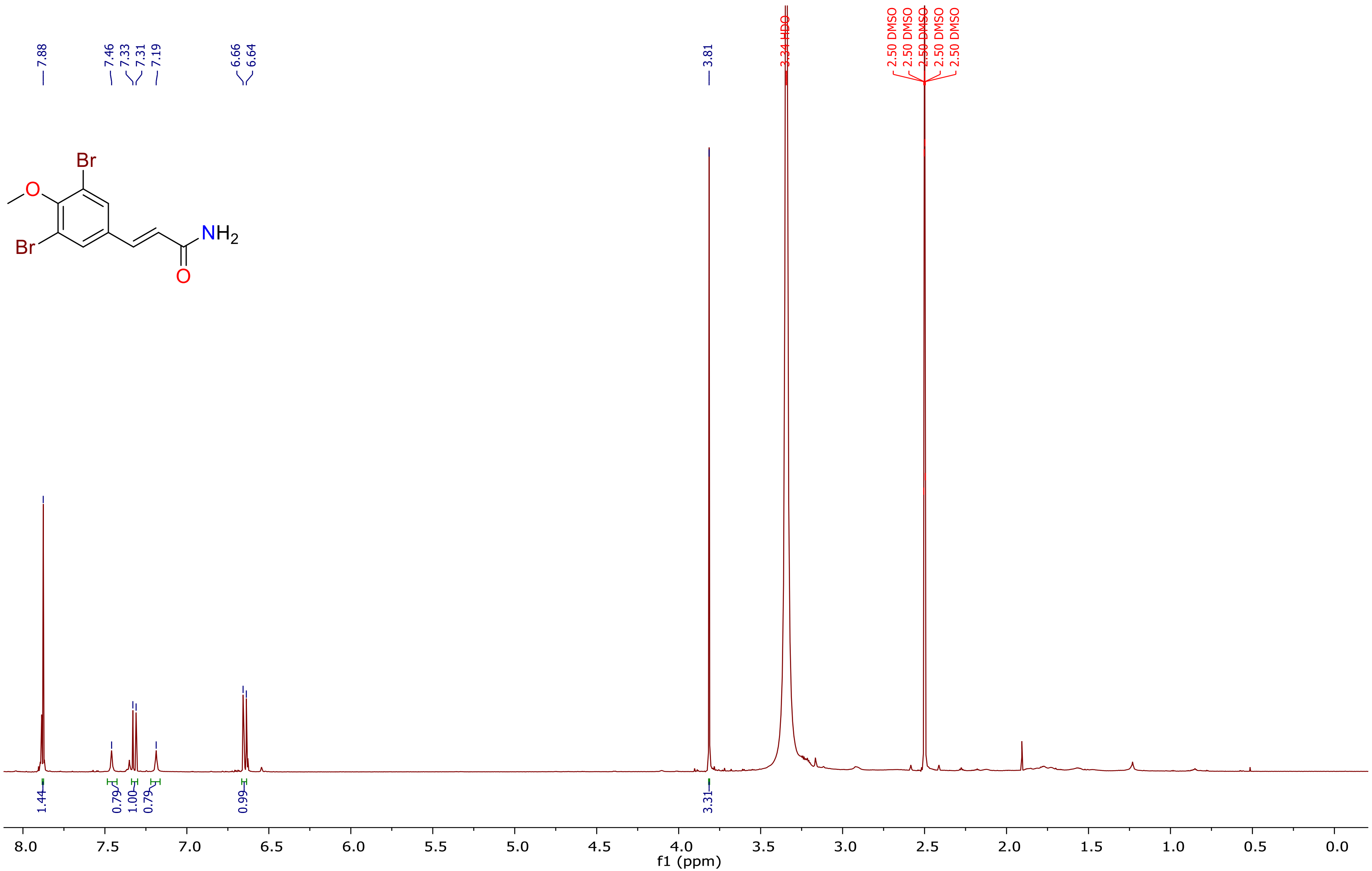

Figure S13: ^{1}H NMR (800 MHz) spectrum of ianthelliformisamine F (**6**) in DMSO-d_6

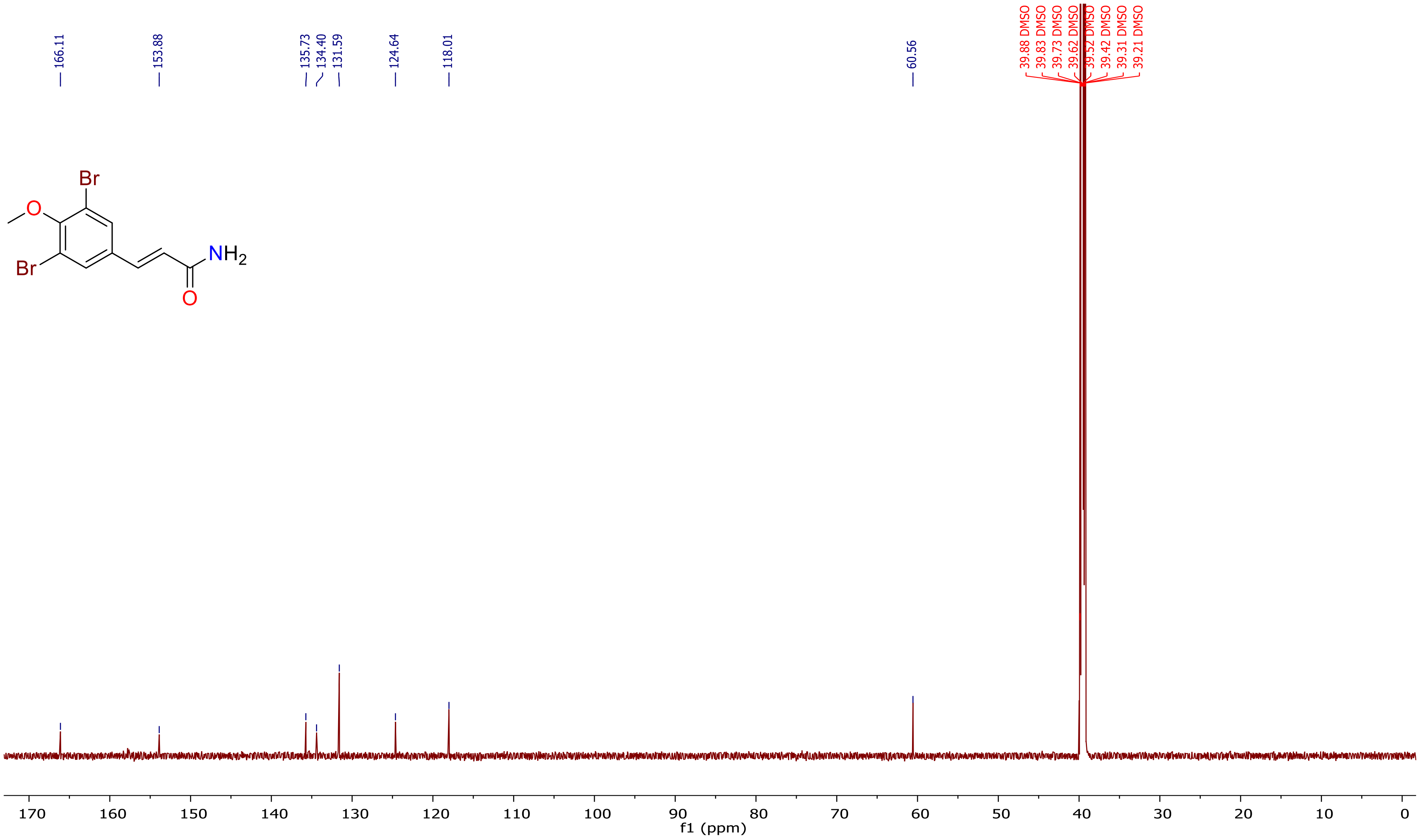

Figure S14: ^{13}C NMR (200 MHz) spectrum of ianthelliformisamine F (**6**) in DMSO-d_6

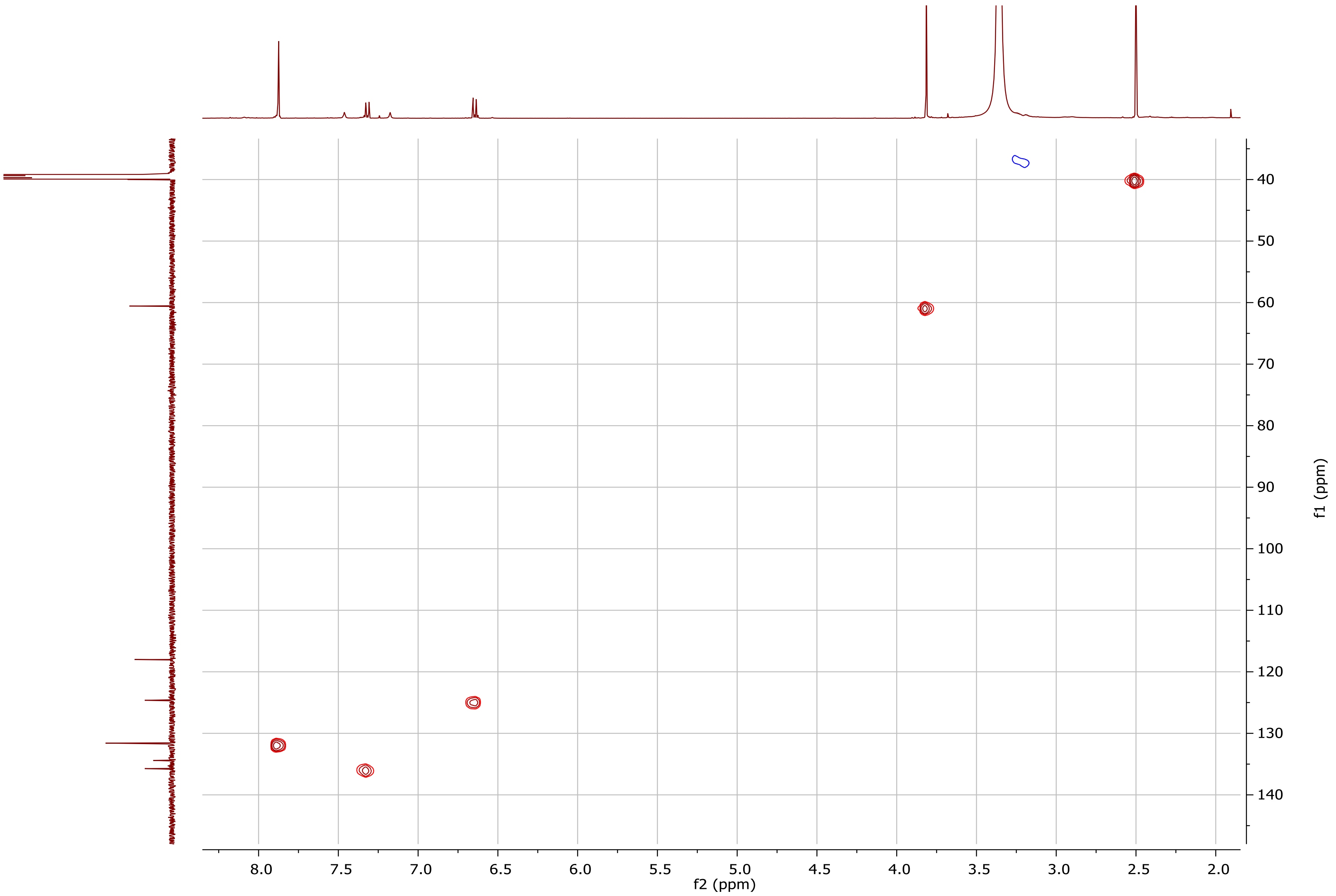

Figure S15: HSQC spectrum of ianthelliformisamine F (**6**) in DMSO-d_6

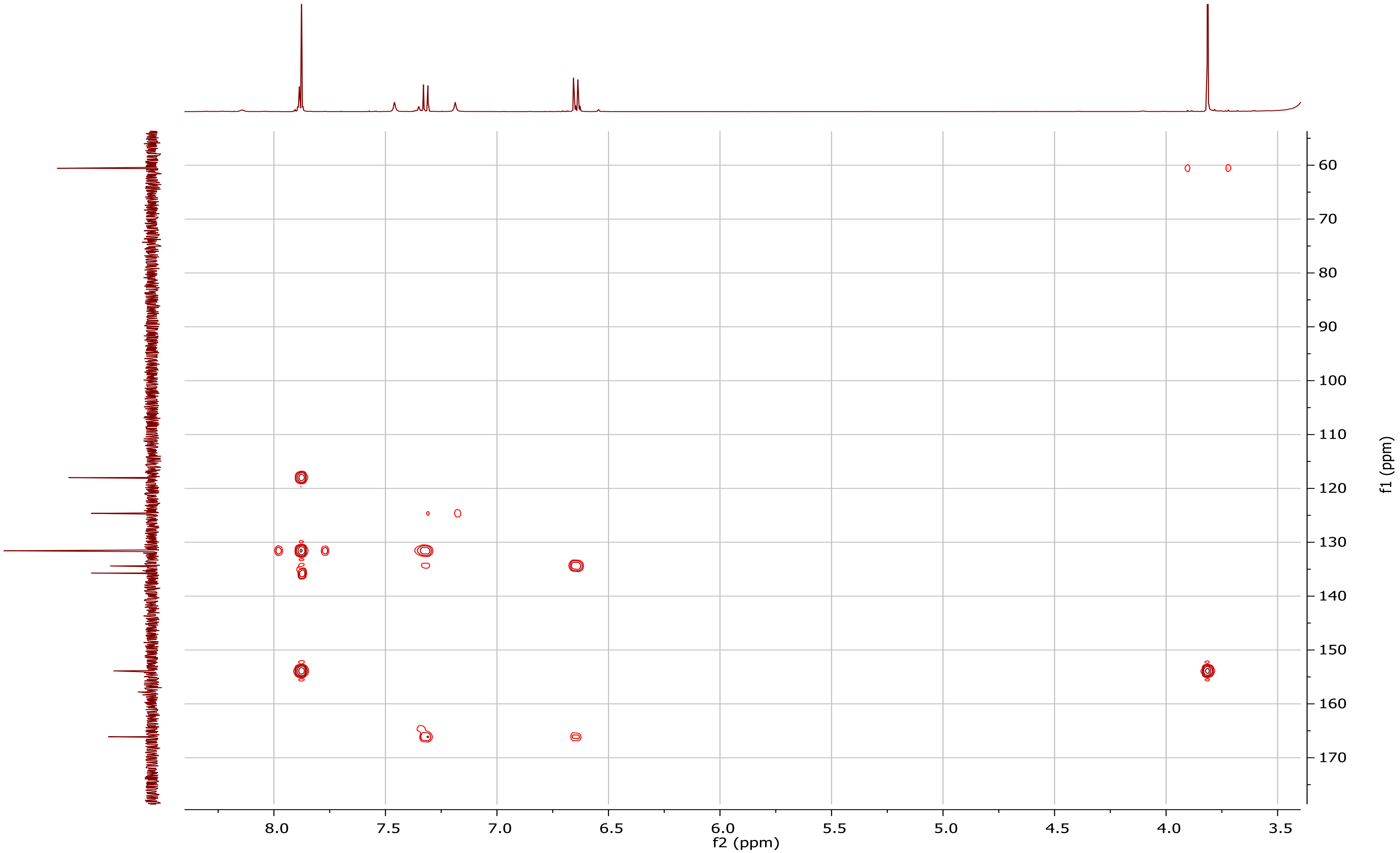

Figure S16: HMBC spectrum of ianthelliformisamine F (**6**) in DMSO-d_6

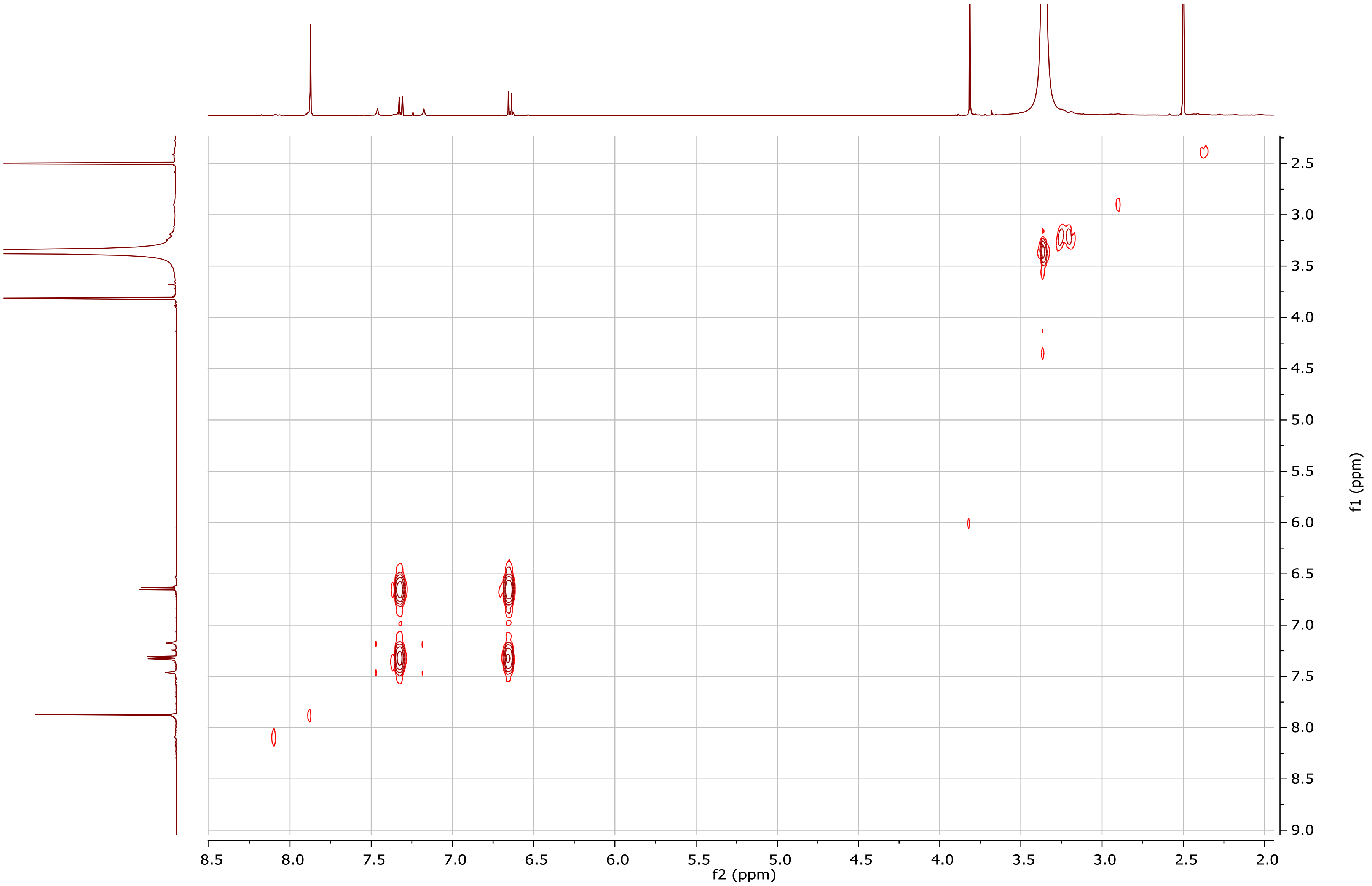

Figure S17: COSY spectrum of ianthelliformisamine F (**6**) in DMSO-d_6

Table S4: NMR data table for ianthelliformisamine G (**7**) in DMSO-d_6[a, b]

position	δ_C, type	δ_H, mult. (J in Hz)	COSY	HMBC
1	134.3, C			
2	131.6, CH	7.89, s		3, 4, 6, 7
3	118.0, C			
4	153.9, C			
4-OCH_3	60.6, CH_3	3.82, s		4
5	118.0, C			
6	131.6, CH	7.89, s		2, 4, 5, 7
7	135.4, CH	7.35, d (15.8)	8	1^w, 2, 6, 8^w, 9
8	124.2, CH	6.65, d (15.8)	7	1, 9
9	164.9, C			
9-NH		8.23, t (5.8)	10	9^w
10	35.9, CH_2	3.25, dt (5.8, 6.8)	9-NH, 11	9, 11^w, 12^w
11	27.5, CH_2	1.73, tt (6.8, 7.5)	10, 12	10, 12
12	36.9, CH_2	2.81, m	11	10^w, 11^w
12-NH_2		7.73, brs		

[a]Spectra recorded at 25 °C (800 MHz for ^{1}H NMR and 200 MHz for ^{13}C NMR); [b]Isolated as a TFA salt; [w]Weak correlation.

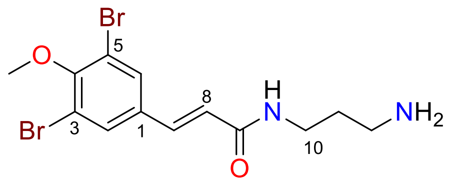

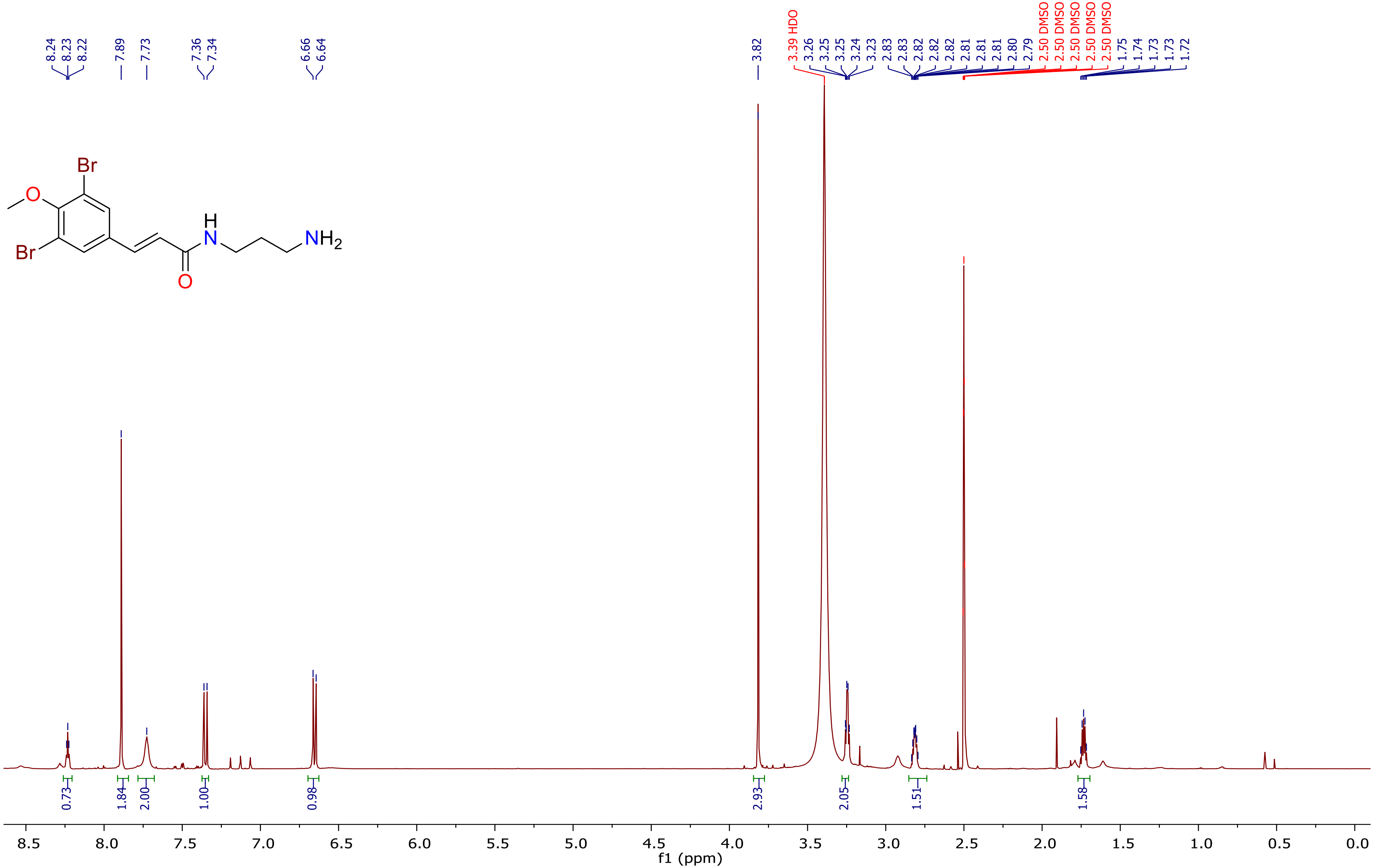

Figure S18: ^{1}H NMR (800 MHz) spectrum of ianthelliformisamine G (**7**) in DMSO-d_6

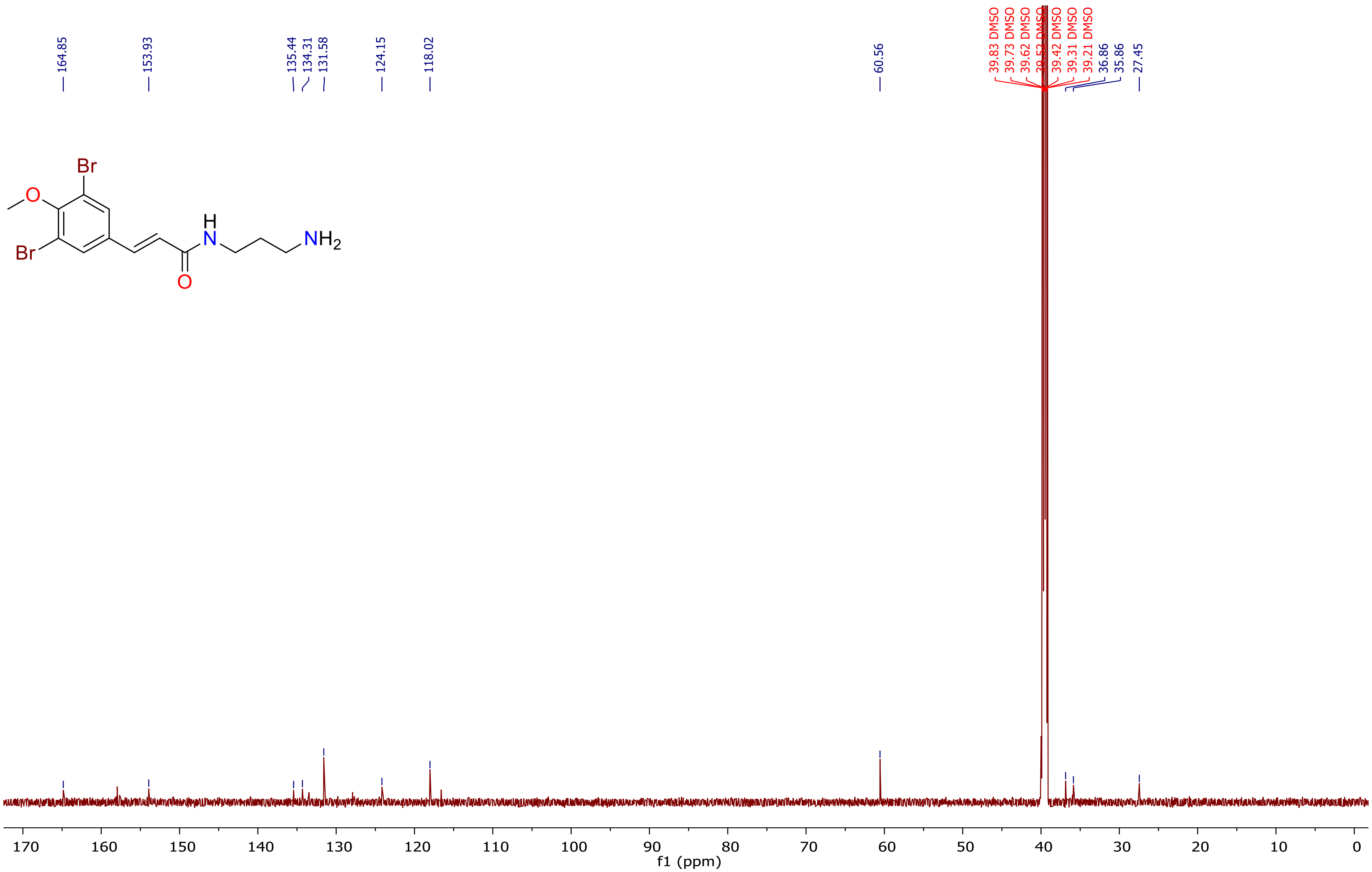

Figure S19: ^{13}C NMR (200 MHz) spectrum of ianthelliformisamine G (**7**) in DMSO-d_6

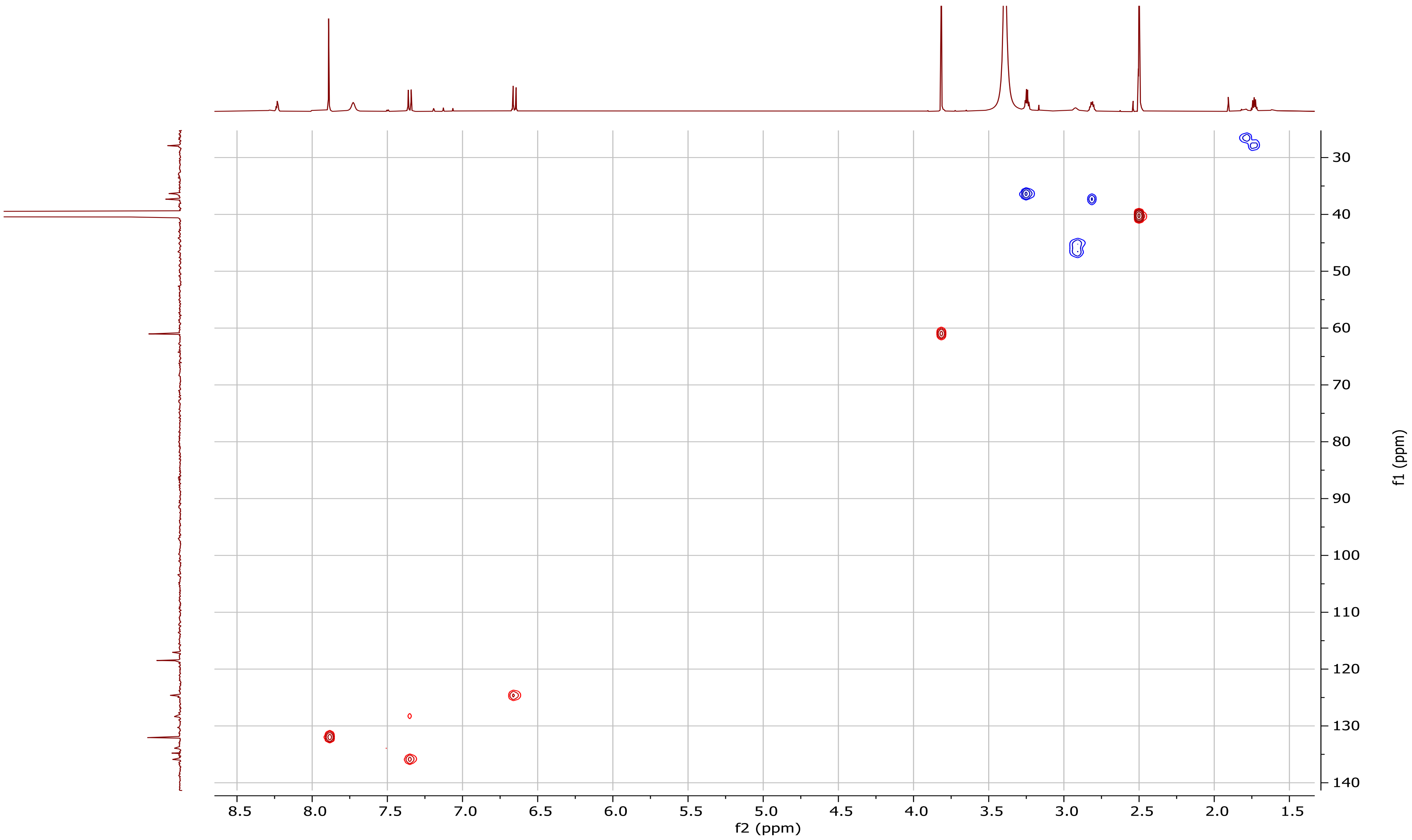

Figure S20: HSQC spectrum of ianthelliformisamine G (**7**) in DMSO-d_6

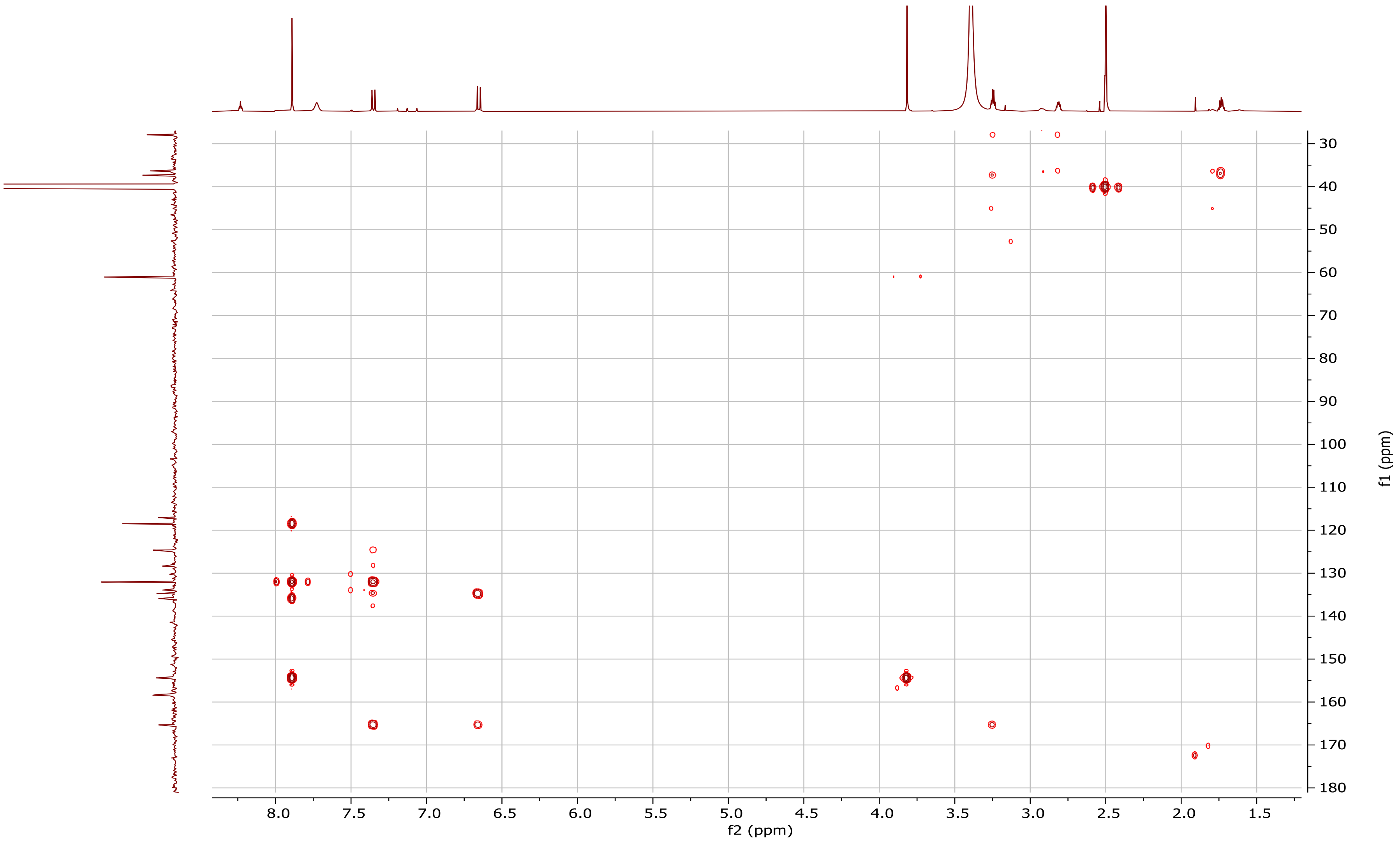

Figure S21: HMBC spectrum of ianthelliformisamine G (**7**) in DMSO-*d*$_6$

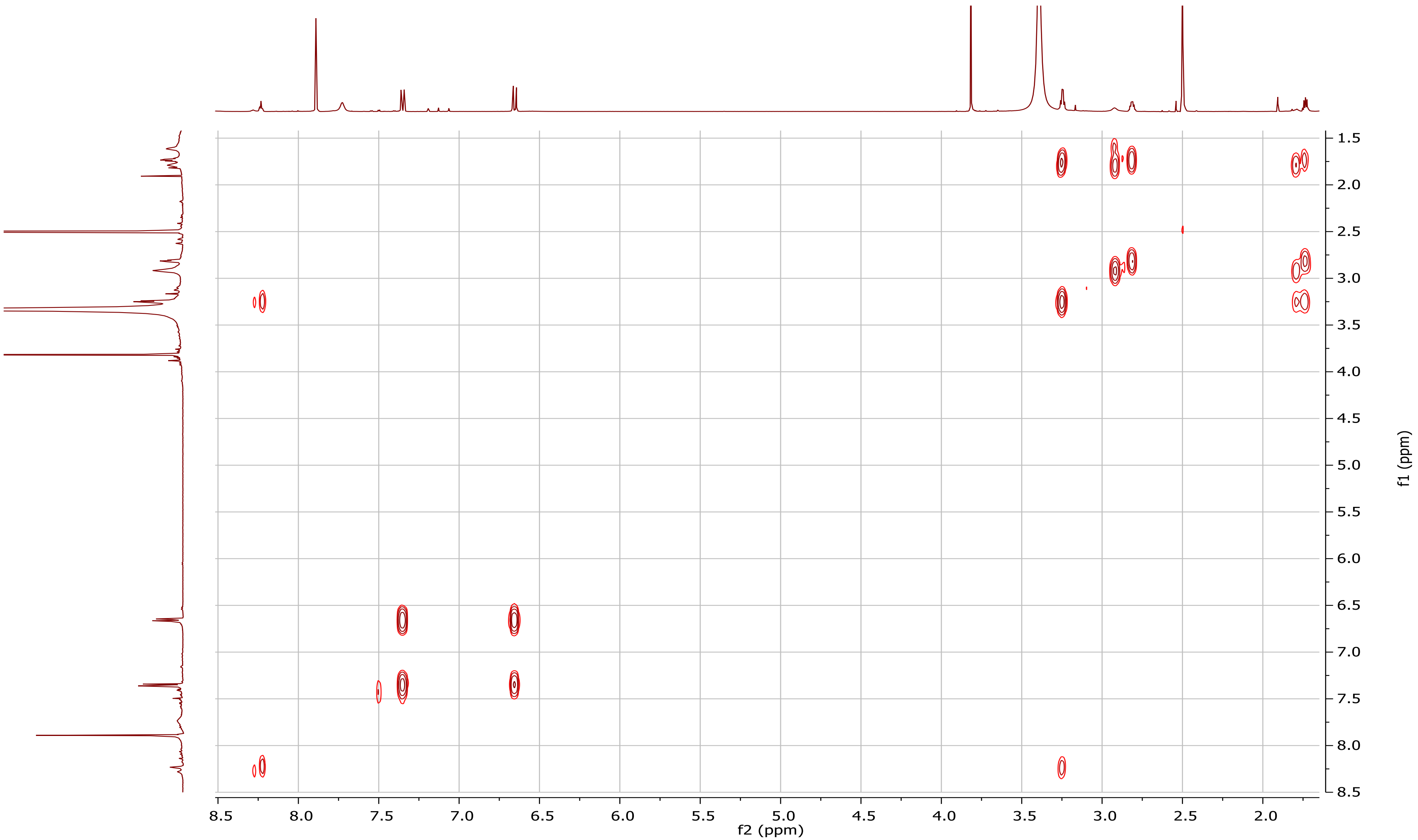

Figure S22: COSY spectrum of ianthelliformisamine G (**7**) in DMSO-d_6

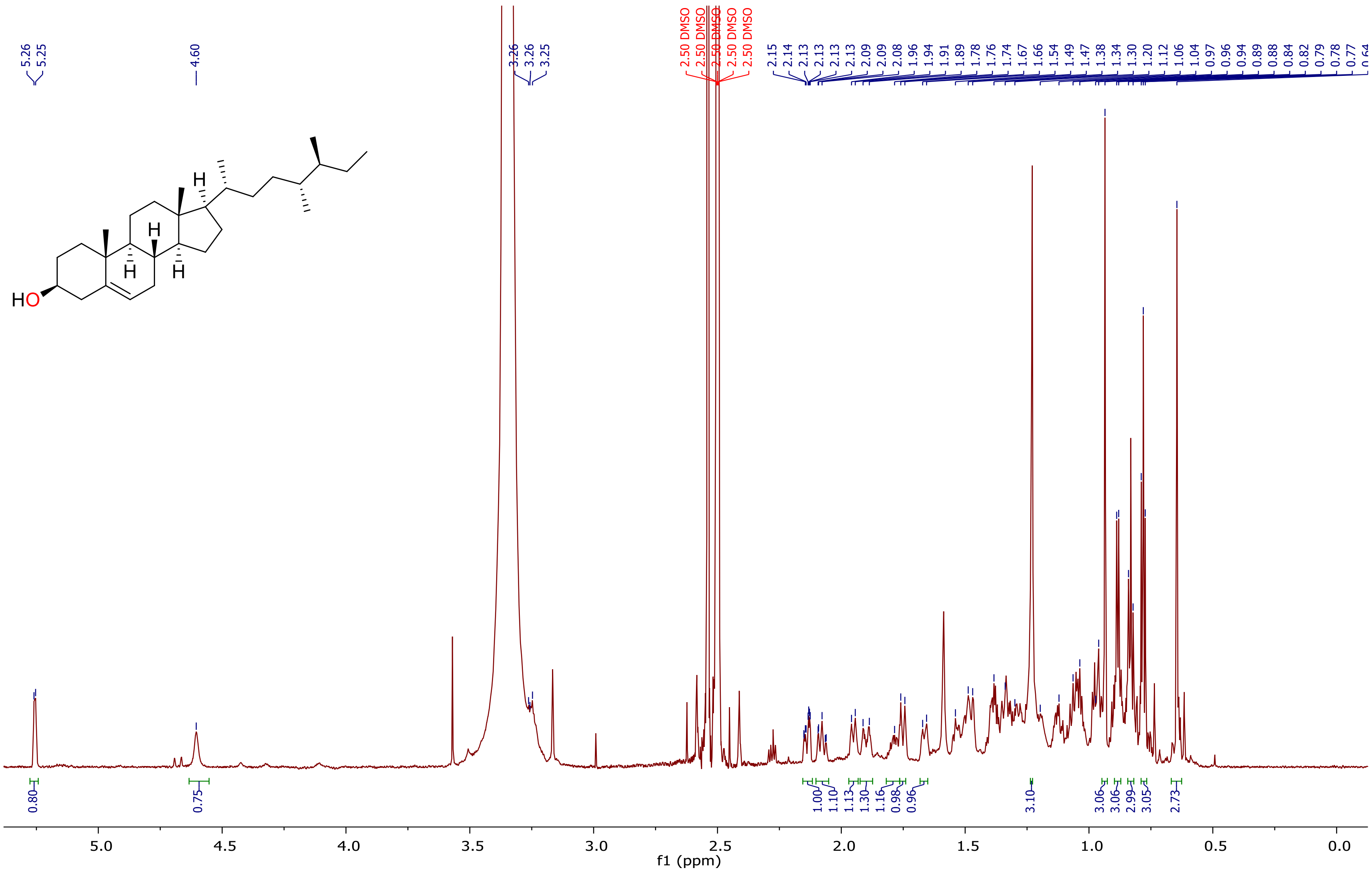

Figure S23: ^{1}H NMR (800 MHz) spectrum of aplysterol (**8**) in DMSO-d_6

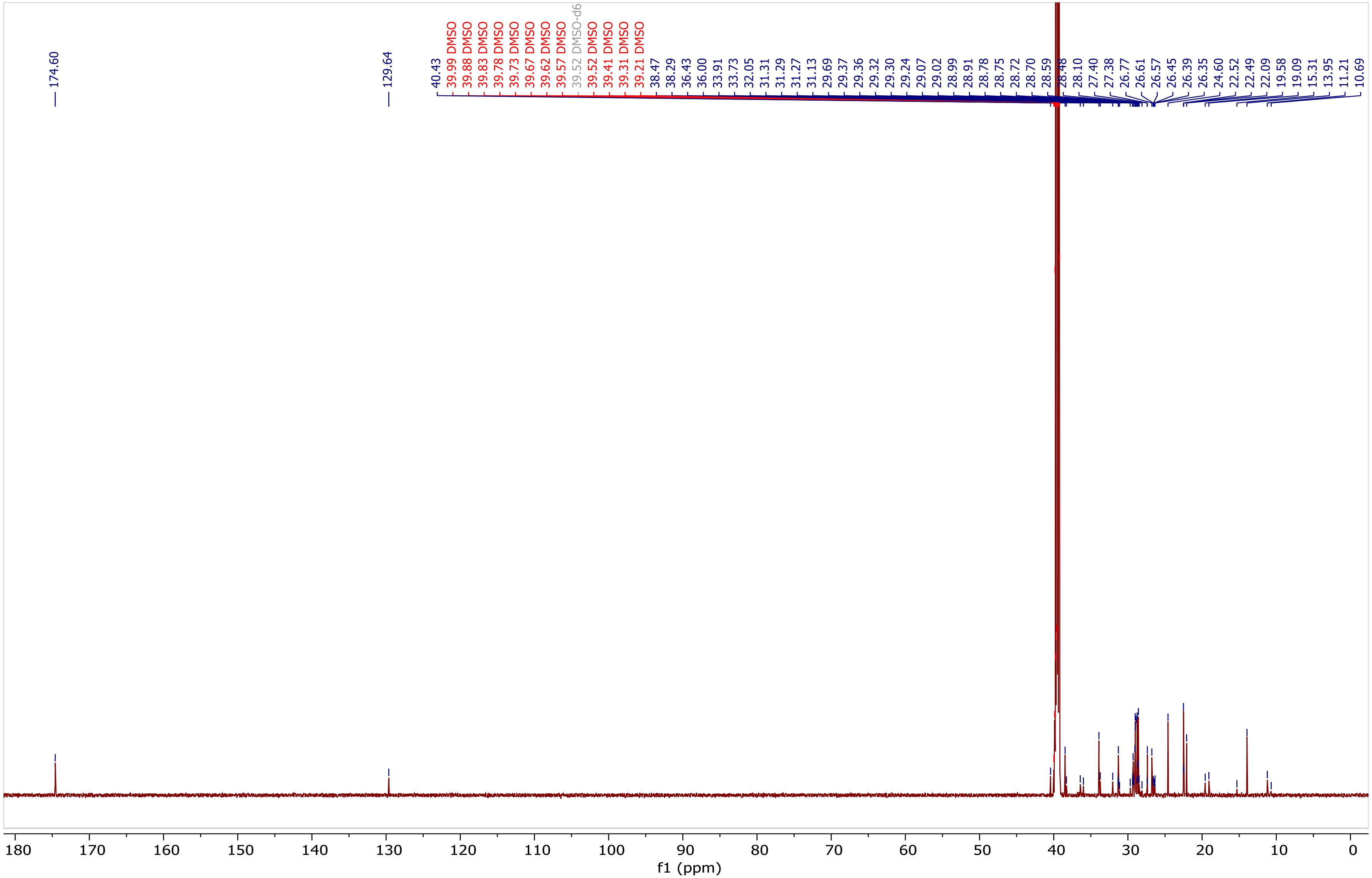

Figure S24: ^{13}C NMR (200 MHz) spectrum of aplysterol (**8**) in DMSO-d_6

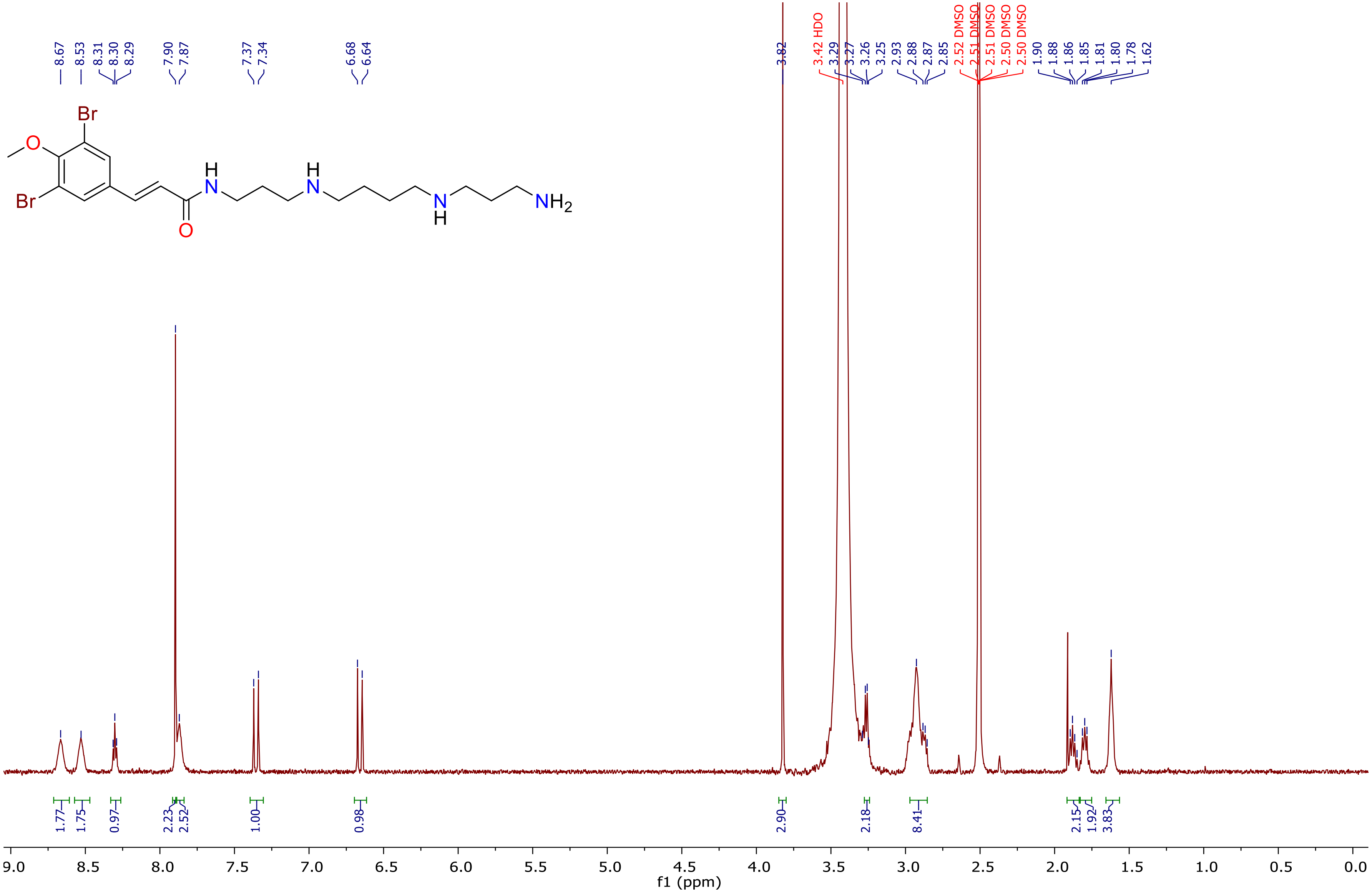

Figure S25: ^{1}H NMR (800 MHz) spectrum of ianthelliformisamine A (**1**) in DMSO-d_6

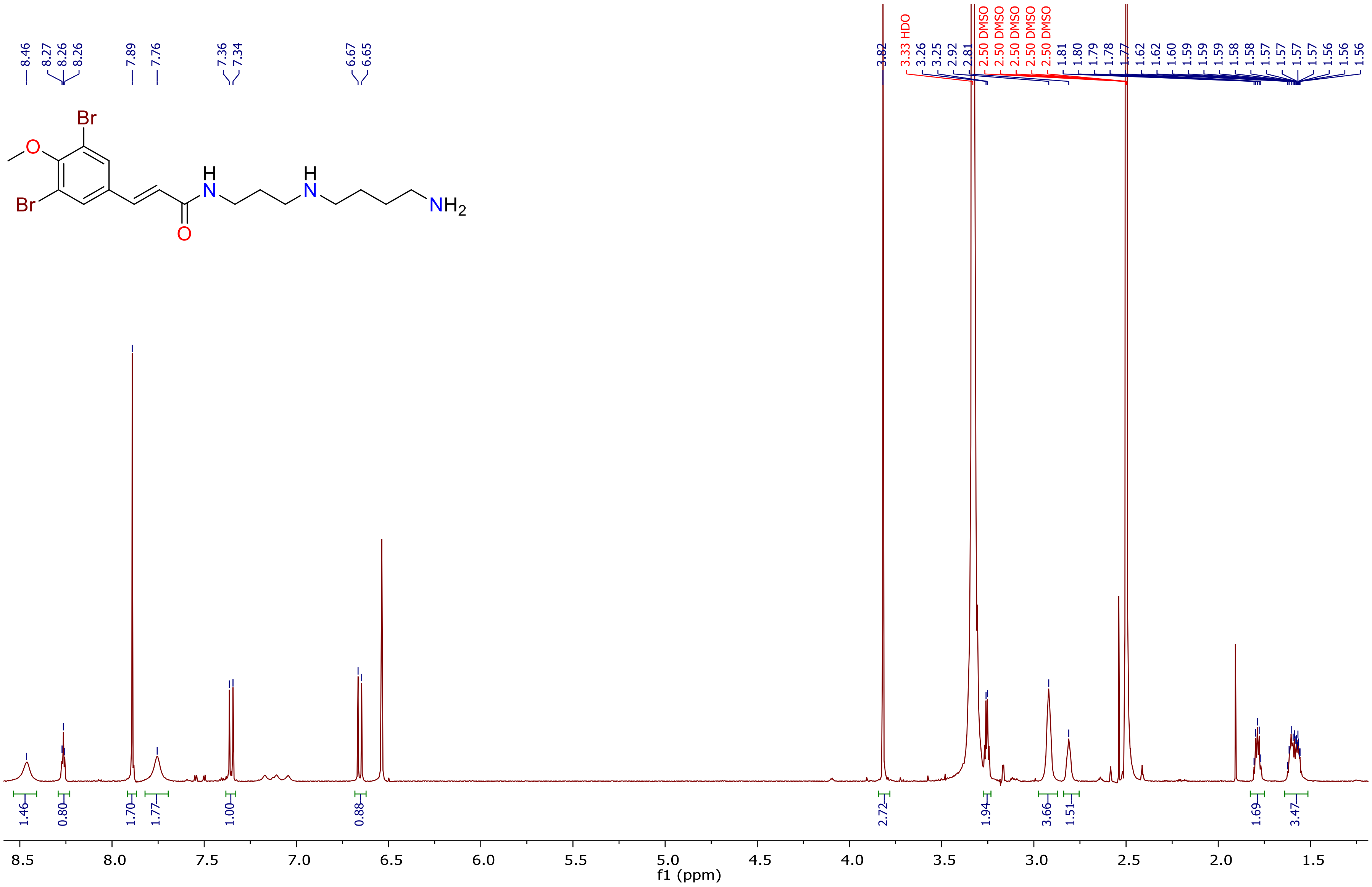

Figure S26: ^{1}H NMR (800 MHz) spectrum of ianthelliformisamine B (**2**) in DMSO-d_6

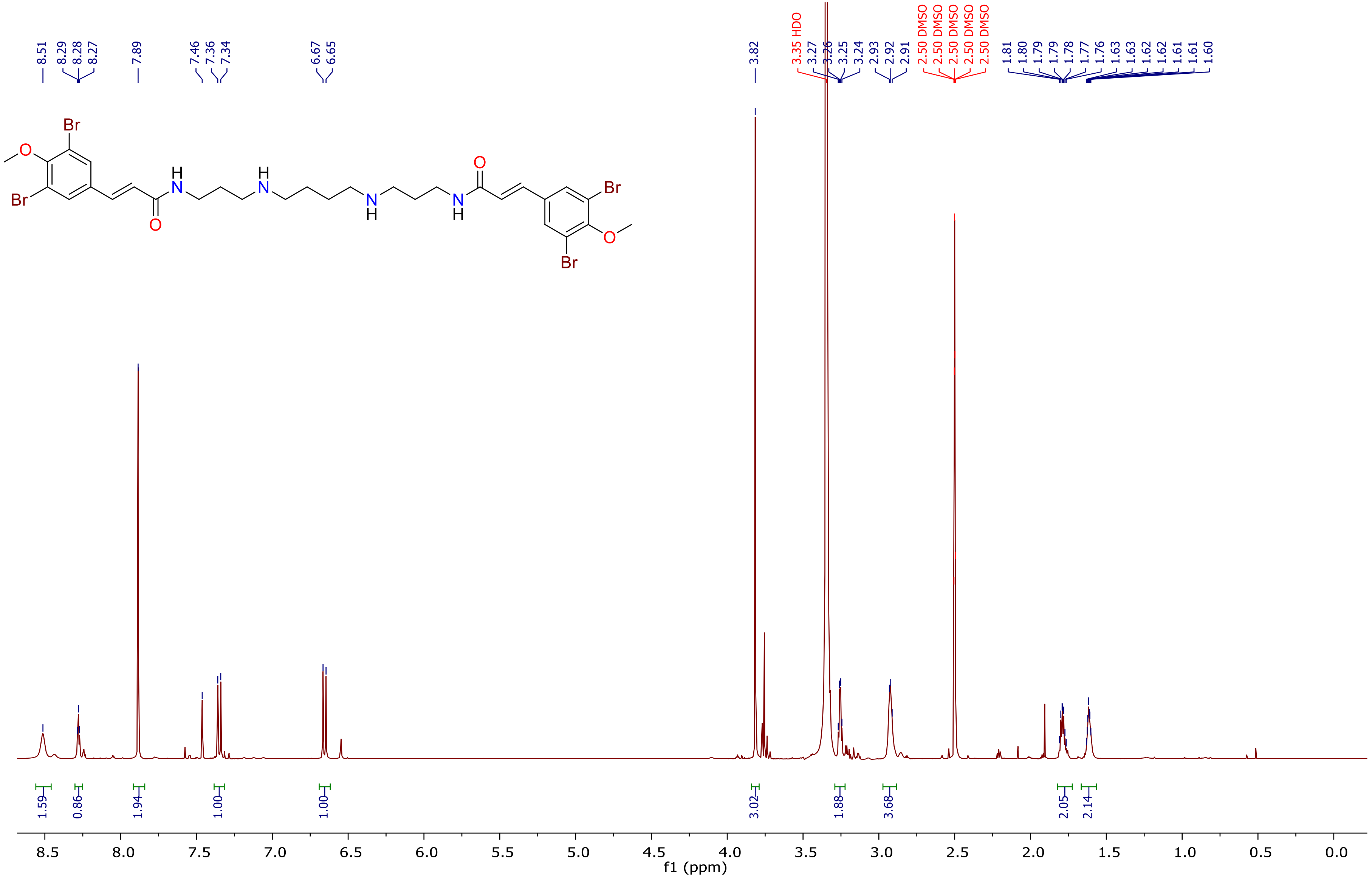

Figure S27: ^{1}H NMR (800 MHz) spectrum of ianthelliformisamine C (**3**) in DMSO-d_6

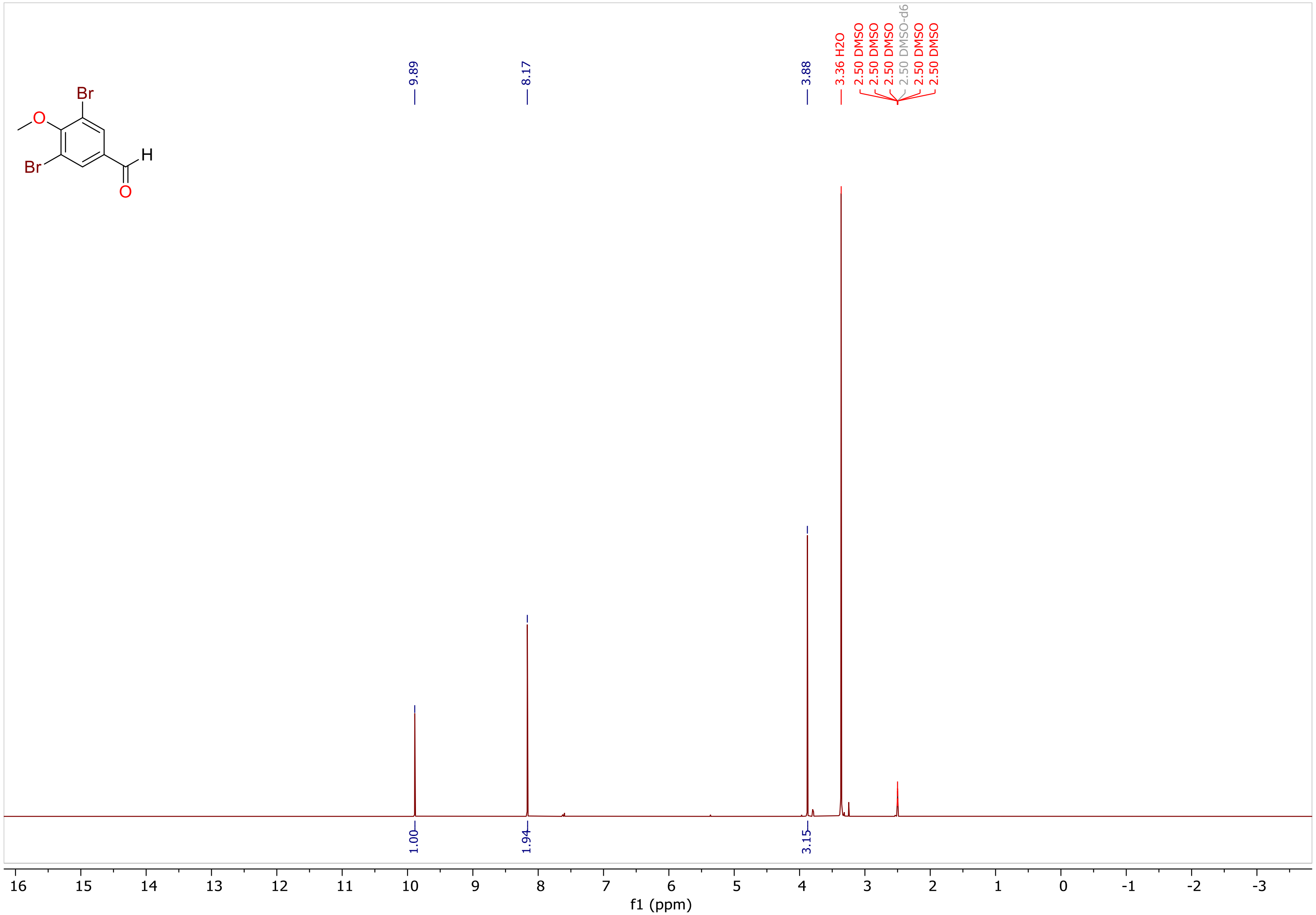

Figure S28: ^{1}H NMR (800 MHz) spectrum of 3,5-dibromo-4-methoxybenzaldehyde (**9**) in DMSO-d_6

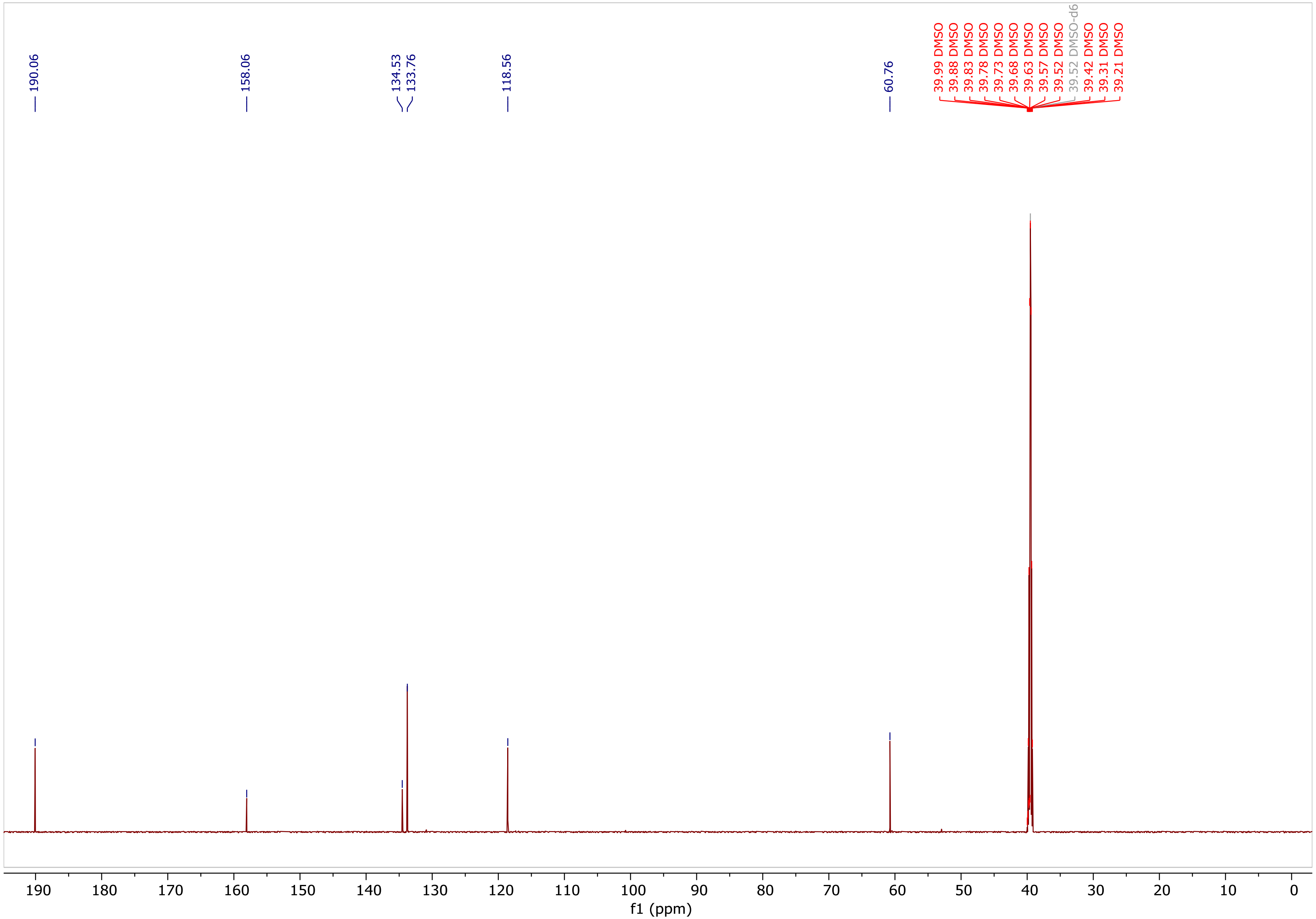

Figure S29: ^{13}C NMR (200 MHz) spectrum of 3,5-dibromo-4-methoxybenzaldehyde (**9**) in DMSO-d_6

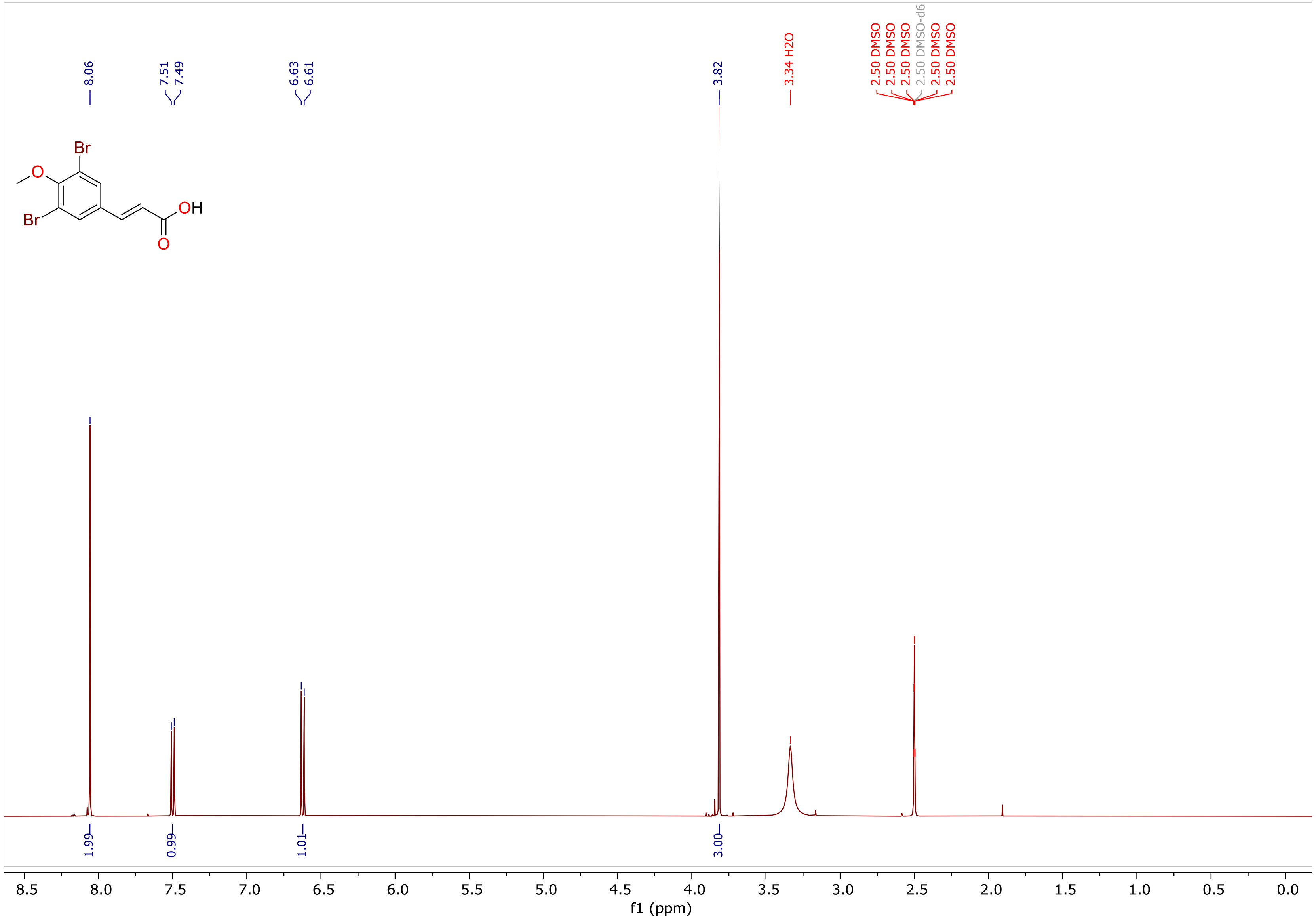

Figure S30: ^{1}H NMR (800 MHz) spectrum of (*E*)-3-(3,5-dibromo-4-methoxyphenyl)acrylic acid (**10**) in DMSO-d_6

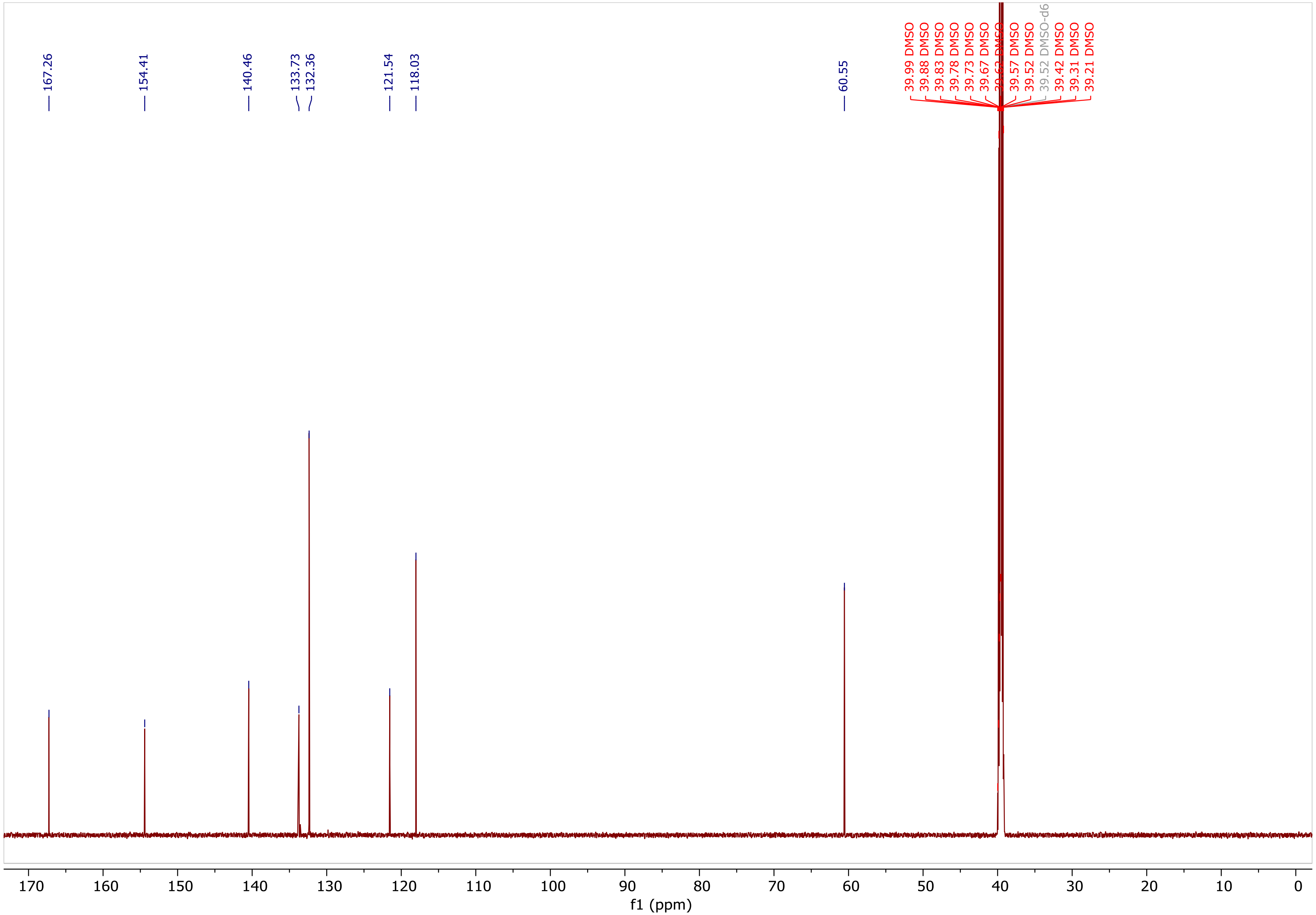

Figure S31: ^{13}C NMR (200 MHz) spectrum of (*E*)-3-(3,5-dibromo-4-methoxyphenyl)acrylic acid (**10**) in DMSO-d_6

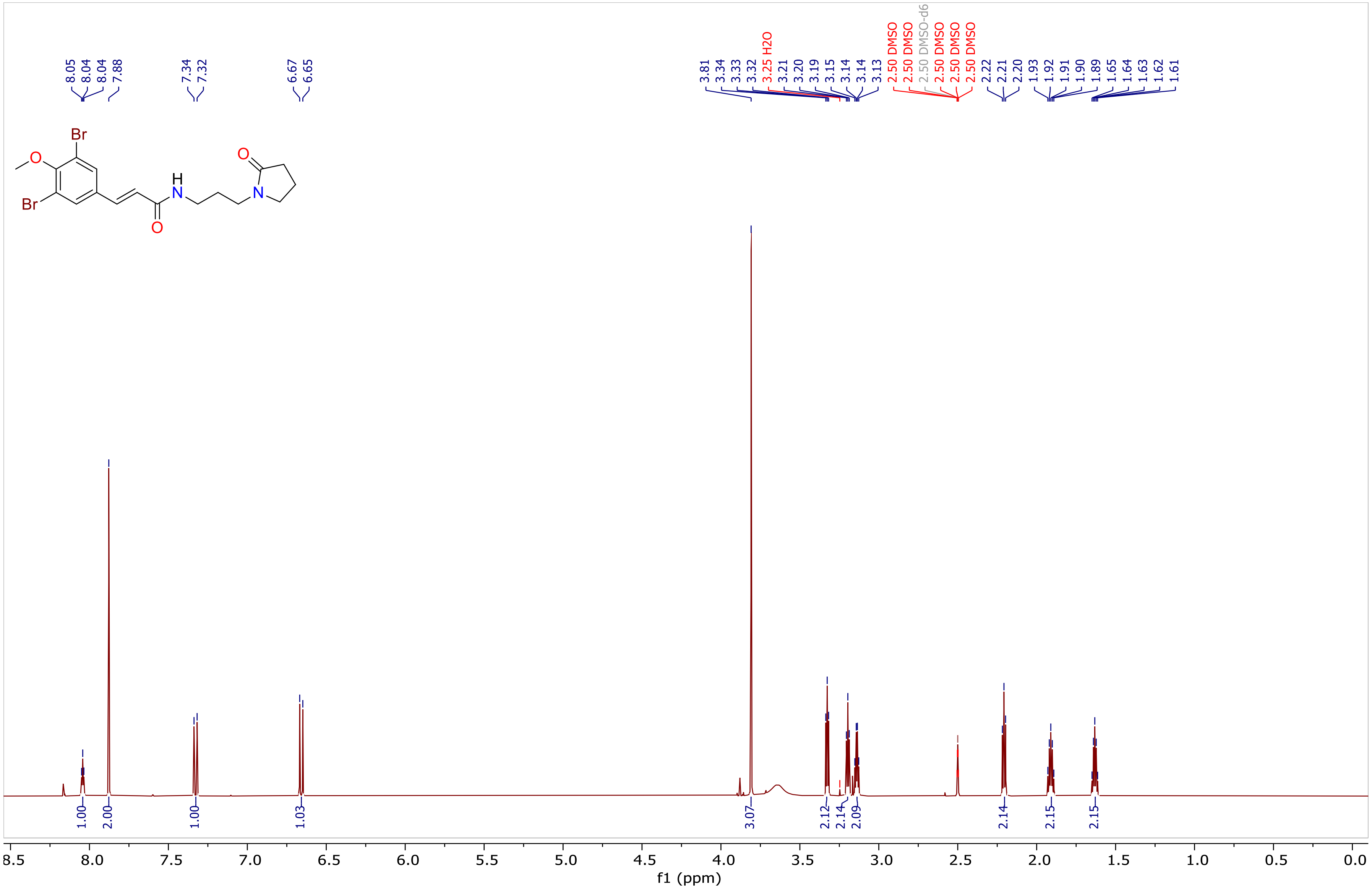

Figure S32: ^{1}H NMR (800 MHz) spectrum of synthetic ianthelliformisamine D (**4**) in DMSO-d_6

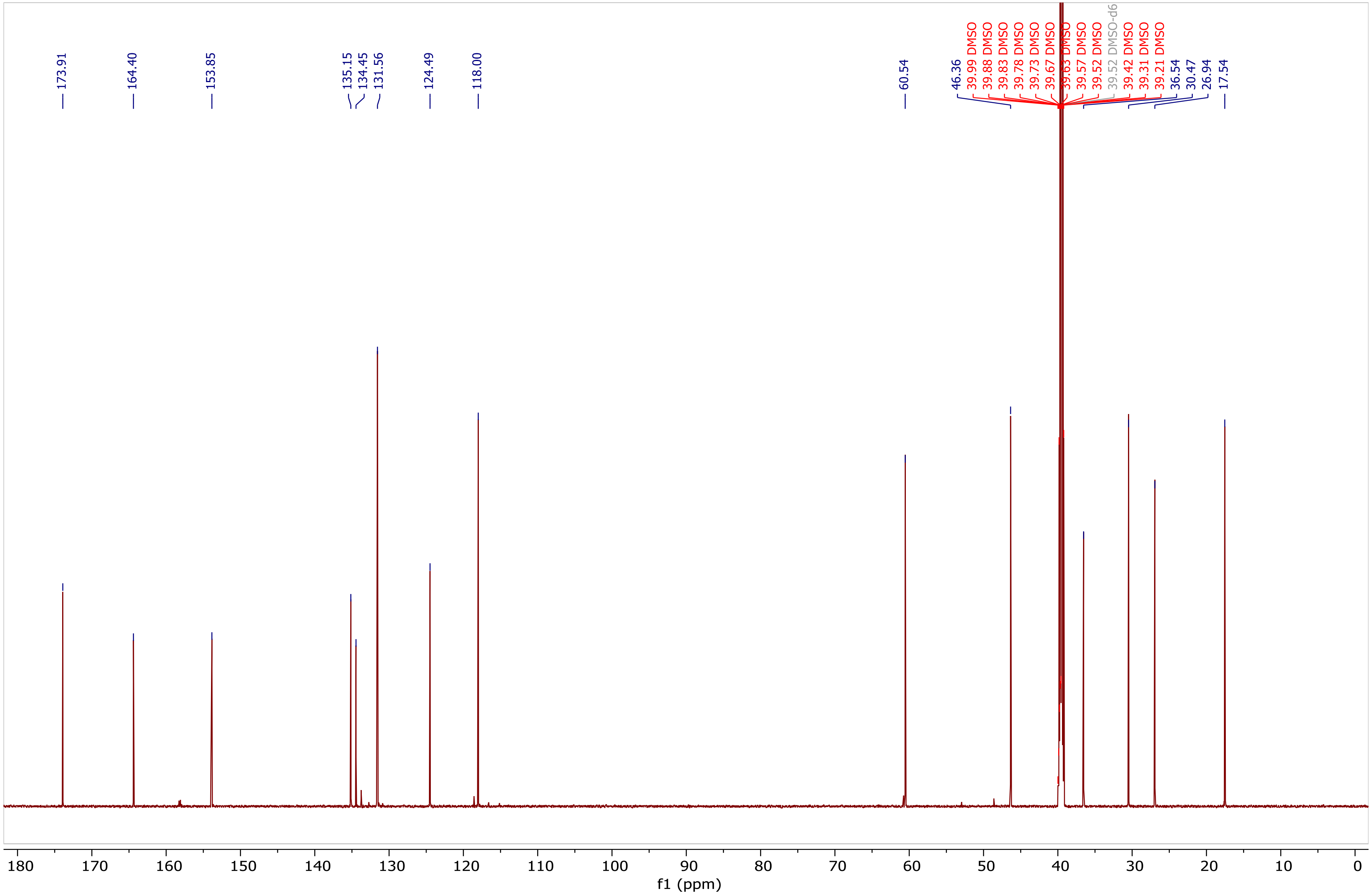

Figure S33: ^{13}C NMR (200 MHz) spectrum of synthetic ianthelliformisamine D (**4**) in DMSO-d_6

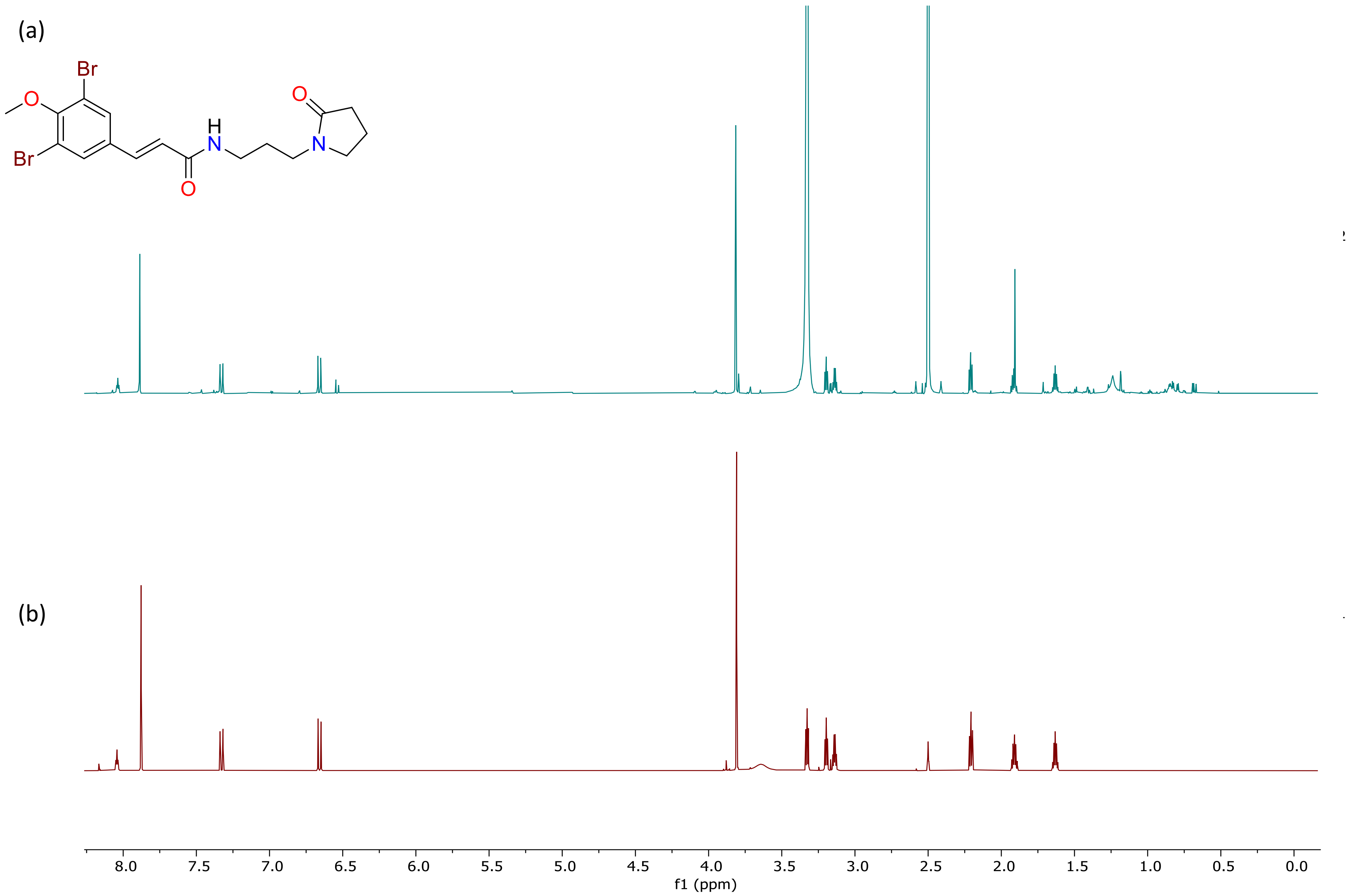

Figure S34: Stacked ^{1}H NMR (800 MHz) spectra of natural product (a) and synthetic (b), ianthelliformisamine D (**4**) in DMSO-d_6

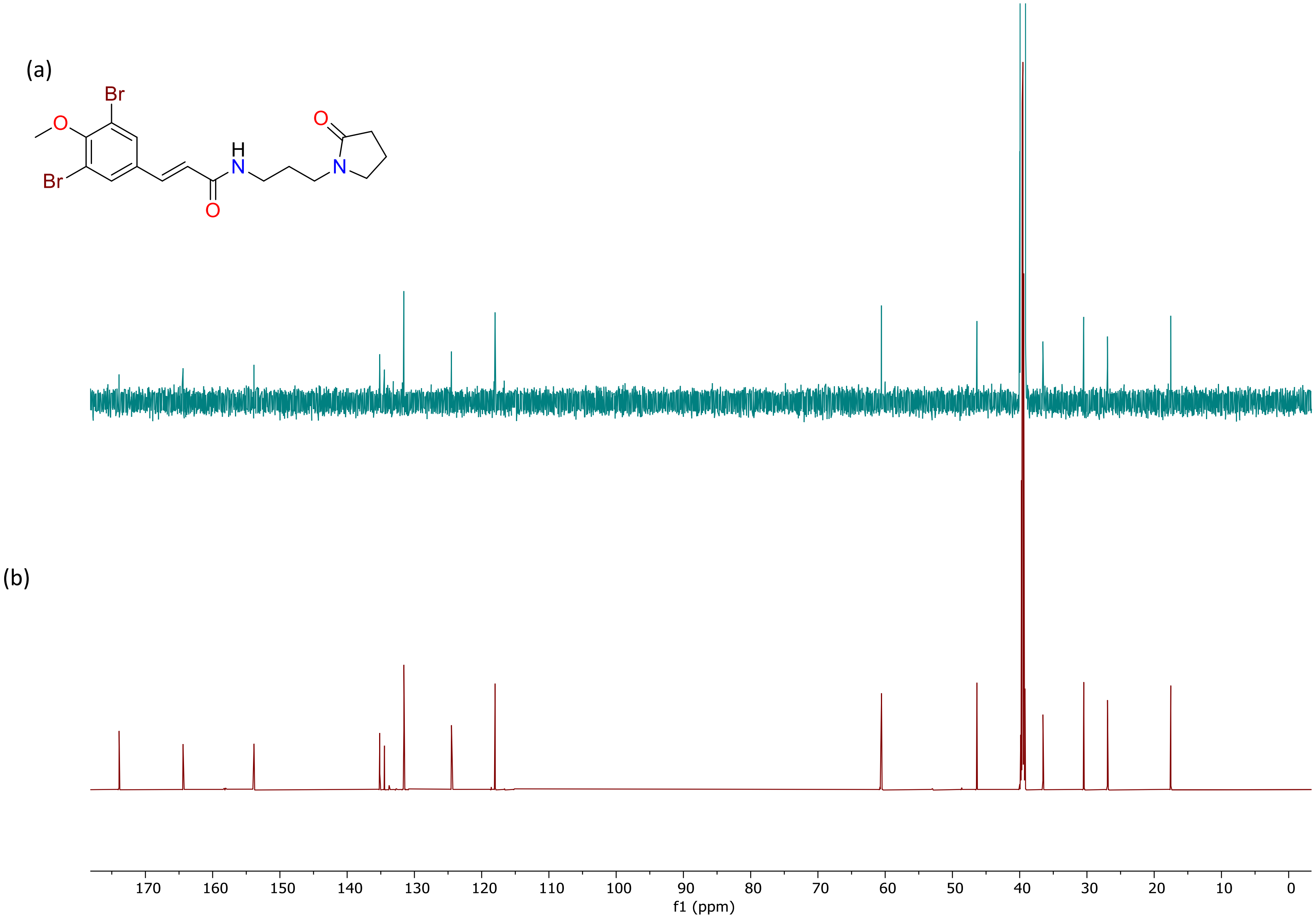

Figure S35: Stacked ^{13}C NMR (200 MHz) spectra of natural product (a) and synthetic (b), ianthelliformisamine D (**4**) in DMSO-d_6
